# Novel chalcone/aryl carboximidamide hybrids as potent anti-inflammatory via inhibition of prostaglandin E2 and inducible NO synthase activities: design, synthesis, molecular docking studies and ADMET prediction

**DOI:** 10.1080/14756366.2021.1929201

**Published:** 2021-05-24

**Authors:** Tarek S. Ibrahim, Amr H. Moustafa, Ahmad J. Almalki, Rasha M. Allam, Abdulhamid Althagafi, Shadab Md, Mamdouh F. A. Mohamed

**Affiliations:** a Department of Pharmaceutical Chemistry, Faculty of Pharmacy, King Abdulaziz University, Jeddah, Saudi Arabia; b Department of Pharmaceutical Organic Chemistry, Faculty of Pharmacy, Zagazig University, Zagazig, Egypt; c Department of Chemistry, Faculty of Science, Sohag University, Sohag, Egypt; d Pharmacology Department, National Research Centre, Cairo, Egypt; e Department of Pharmacy Practice, Faculty of Pharmacy, King Abdulaziz University, Jeddah, Saudi Arabia; f Department of Pharmaceutics, Faculty of Pharmacy, King Abdulaziz University, Jeddah, Saudi Arabia; g Department of Pharmaceutical Chemistry, Faculty of Pharmacy, Sohag University, Sohag, Egypt

**Keywords:** Chalcone, carboximidamide, iNOS inhibitors, anti-inflammatory, amidoxime, PGE2

## Abstract

Two series of chalcone/aryl carboximidamide hybrids **4a–f** and **6a–f** were synthesised and evaluated for their inhibitory activity against iNOS and PGE2. The most potent derivatives were further checked for their *in vivo* anti-inflammatory activity utilising carrageenan-induced rat paw oedema model. Compounds **4c**, **4d**, **6c** and **6d** were proved to be the most effective inhibitors of PGE2, LPS-induced NO production, iNOS activity. Moreover, **4c**, **4d**, **6c** and **6d** showed significant oedema inhibition ranging from 62.21% to 78.51%, compared to indomethacin (56.27 ± 2.14%) and celecoxib (12.32%). Additionally, **4c**, **6a** and **6e** displayed good COX2 inhibitory activity while **4c**, **6a** and **6c** exhibited the highest 5LOX inhibitory activity. Compounds **4c**, **4d**, **6c** and **6d** fit nicely into the pocket of iNOS protein (PDB ID: 1r35) *via* the important amino acid residues. Prediction of physicochemical parameters exhibited that **4c**, **4d**, **6c** and **6d** had acceptable physicochemical parameters and drug-likeness. The results indicated that chalcone/aryl carboximidamides **4c**, **4d**, **6c** and **6d**, in particular **4d** and **6d**, could be used as promising lead candidates as potent anti-inflammatory agents.

## 1. Introduction

Inflammation is a complex process in the host defence mechanism for the protection against injuries, microbial infections, and foreign substances^1^^,^^2^. It includes various cellular and plasma regulators that limit its action at a critical time and place^3^. There are two types of inflammation, namely, acute and chronic inflammation. If the acute inflammation persists for a long time, it may lead to systemic or chronic serious inflammatory disorders that results in several damaging consequences on the host cells and tissues and consequently leading to the development of cardiovascular diseases and cancer^3^. Upon inflammatory stimulation, macrophages generate a diversity of inflammatory mediators such as prostaglandin E_2_ (PGE_2_) and nitric oxide (NO)^4^. PGE2 is an influential lipid inflammatory mediator produced *via* the COX pathway and distributed in human body^5^^,^^6^. PGE2 is the most copious metabolic product which is responsible for the inflammatory-related disorders and shows a crucial role in vascular permeability, hyperalgesia and pyresis^7^. Moreover, nitric oxide (NO), an important free radical key inflammatory mediator in living organisms that has vital functions in the physiological and pathophysiological regulation mechanisms at cardiovascular, nervous and immunological systems^8^. The NO overproduction might responsible for the immunological pathology of macrophage-dependent inflammatory and degenerative diseases, together with cancer^9^. In mammals, NO is produced *via* l-arginine oxidation by NO synthase (NOS); additionally, there are three NOS enzyme isoforms have been detected: endothelial NOS, neuronal NOS and inducible NOS (iNOS)^10^^,^^11^. Consequently, inhibition of iNOS-mediated NO and PGE_2_ generation is a favourable therapeutic target in the discovery of effective anti-inflammatory agents for the therapy of inflammatory diseases^10^.

Notably, prolonged use of traditional non-steroidal anti-inflammatory drugs (NSAIDs), e.g. aspirin, indomethacin and ibuprofen promoted the appearance of several undesired side effects such as gastrointestinal irritation, ulceration and bleeding due to their inhibitory action on the gastroprotective prostanoids formed by COX-1 enzymes in the gastrointestinal tract^3^^,^^12^ that highlighted the significance of the innovation of new potent and selective COX-2 inhibitors with less gastro-intestinal side effects and with more safety profile such as celecoxib and its analogues^13^^,^^14^. Nevertheless, prolonged administration of selective COX-2 for inhibition of prostaglandins production stimulated the metabolism of arachidonic acid pathway to LOX enzyme leading to increased availability of substrate which consequently resulted in increased leukotriene production by lipoxygenase pathway that intensifies airway inflammation and exaggerates bronchoconstriction^12^. Thus, efforts have been focussed on the design and innovation of novel anti-inflammatory drugs with minimum or no side effects as an alternative to non-selective NSAIDs that might be valuable for the managing of inflammatory diseases^3^^,^^12^.

Chalcone is a type of open chain flavonoids with two aryl rings linked through a three-carbon propanone spacer. The α,β-unsaturated propanone fragment facilitates the conversion of chalcones to several classes of heterocyclic compounds^15^ such as flavonoids, iso flavonoids^16^, pyrazoles^17^, 2-pyrazolines^18^^,^^19^, imidazoles and pyrimidines^20^. Chalcones and their derivatives have attracted substantial research attention^21^ not only due to their ease of synthesis but also due to their varied and interesting biological activities such as HDAC inhibitory activity^22^, anticancer activities^23^^,^^24^, antihypertensive^25^, antimalarial^26^^,^^27^, antioxidant^28^^,^^29^, analgesic^30^, anti-inflammatory^31^, antibacterial^32^, antifungal^33^^,^^34^, antiulcer agents^35^, parasitic protease inhibitors^36^, antiviral^37^, anti-tuberculosis^38^ and as insulin mimetic in 3T3-L1 adipocyte^39^.

**Figure 1.**
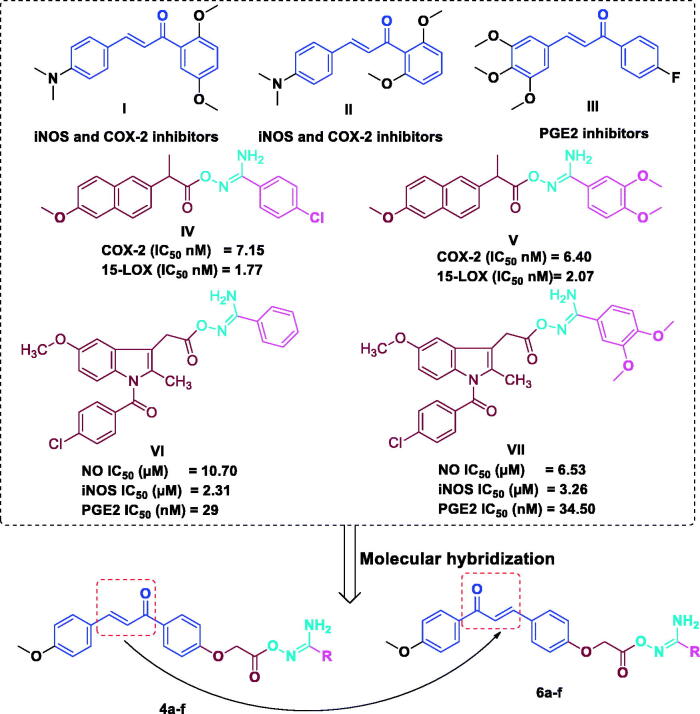
Structures of anti-inflammatory chalcones (**I–III**), aryl carboximidamides (**V–VII**) and rational design of chalcone/aryl carboximidamides as novel iNOS/PGE2 inhibitors.

Moreover, both natural and synthetic chalcones exert their the anti-inflammatory activities (Figure 1) against various therapeutic targets such as cyclooxygenase (COX-1 and COX-2), lipooxygenase (LOX), nitric oxide synthase (NOS), interleukins, expression of cell adhesion molecules (CAM) and prostaglandins (PGs)^40^.

On the other hand, amidoxime derivatives exhibited notable biological activities as anti-inflammatory, antihyperglycemic, antimycobacterial, serotonergic inhibitory, muscarinic agonist and peptide inhibitory activities^12^^,^^41–45^. It has been used as bioisostere for carboxylic and ester groups for the design of drugs having improved pharmacokinetic (PK) and pharmacodynamic (PD) properties^45^. Recently, we have investigated the effects of a series of aryl carboximidamides appended Naproxen derivatives as dual acting COX-2/15-LOX inhibitors^12^ (Figure 1). Among these estimated derivatives, **IV** and **V** were the targets having remarkable inhibitory potencies; furthermore, compound **V** is the most potent as COX-2 inhibitor with approximately 6.6-folds higher than the reference drug, celecoxib and compound **V** has bestowed with the strongest 15-LOX inhibitory activity. Moreover, and very recently we have studied the effects of aryl carboximidamides appended indomethacin as dual iNOS/PGE2 inhibitors^10^. Most of the target indomethacin/aryl carboximidamides displayed powerful inhibitory action against LPS-prompted NO production. Compounds **VI** and **VII** (Figure 1), showed significant *in vivo* anti-inflammatory activity. Notably, compound **VI** demonstrated inhibition to LPS-induced NO production, iNOS activity and PGE2 with IC_50_ of 10.70 nM, 2.31 nM and 29 nM, respectively.

Encouraged by the previously mentioned information, herein, we report the synthesis and the *in vivo* anti-inflammatory screening of two series of chalcone/aryl carboximidamides **4a–f** and **6a–f** (Figure 1) as iNOS and PGE_2_ inhibitors compared to indomethacin as standard well-known NSAID.

## 2. Experimental

### 2.1. Chemistry

#### 2.1.1. General details (see Supporting information)

##### 2.1.1.1. General procedure for the synthesis of N’-{2-[1-(4-chlorobenzoyl)-5-methoxy-2-methyl-1H-indol-3-yl]acetoxy}arylcarboximidamide (4a–f)

To a suspension of chalcones **3** or **5** (1.5 mmole) in 30 ml acetonitrile, **CDI** (1.8 mmole) was added and the mixture was allowed to stir at room temperature for 30–60 min. Then, amidoximes **2a–f** (1.5 mmole) was added and stirring continued for further 3 h. After completion of the reaction (as monitored by TLC), the formed precipitate was collected by filtration, washed several times with cold acetonitrile, dried and recrystallized from acetonitrile to afford **4a–f** and **6a–f**.

##### 2.1.1.2. *N’*-(2-{4-[(2*E*)-3-(4-Methoxyphenyl)prop-2-enoyl]phenoxy}acetoxy) benzenecarboximidamide (4a)

Yield 86%; white solid; m.p.: 164–166 °C. IR (ATR) *ν*_max_ 3444, 3322 (NH_2_), 3067 (C–H aromatic), 2996, 2967, 2834 (C–H aliphatic), 1755 (C=O), 1651 (C=O), 1627 (C=N) cm^−1^. ^1^H NMR (400 MHz, DMSO-d_6_) *δ* 3.83 (s, 3H, OCH_3_), 5.15 (s, 2H, CH_2_), 6.96 (s, 2H, NH_2_), 7.02 (d, *J* = 8.4 Hz, 2H, CH_arom._), 8.15 (d, *J* = 8.6 Hz, 2H, CH_arom._), 7.45–7.53 (m, 3H, CH_arom._), 7.68–7.85 (m, 6H, CH_arom._), 8.16 (d, *J* = 8.6 Hz, 2H, CH_arom._); ^13^C NMR (100 MHz, DMSO-d_6_) *δ* 55.8, 64.8, 114.9, 115.0, 120.1, 127.3, 127.6, 127.9, 128.9, 129.8, 131.1, 131.7, 131.8, 143.7, 158.1, 161.7, 161.9, 167.2, 187.9; -ESI-MS (*m*/*z*): 429.4, [M-H]^−^; Anal. Calcd. for C_25_H_22_N_2_O_5_ (430.45): C, 69.76; H, 5.15; N, 6.51. Found: C, 69.58; H, 5.25; N, 6.38.

##### 2.1.1.3. *N’*-(2-{4-[(2*E*)-3-(4-Methoxyphenyl)prop-2-enoyl]phenoxy}acetoxy)-4-chlorobenzenecarboximidamide (4b)

Yield 88%; white solid; m.p.: 180–182 °C. IR (ATR) *ν*_max_ 3508, 3379 (NH_2_), 3073, 3052 (C–H aromatic), 2998, 2928, 2833 (C–H aliphatic), 1763 (C=O), 1654 (C=O), 1621 (C=N) cm^−1^. ^1^H NMR (400 MHz, DMSO-d_6_) *δ* 3.83 (s, 3H, OCH_3_), 5.14 (s, 2H, CH_2_), 7.2 (d, *J* = 8.4 Hz, 4H, NH_2_ + CH_arom._), 7.14 (d, *J* = 8.7 Hz, 2H, CH_arom._), 7.55 (d, *J* = 8.4 Hz, 2H, CH_arom._), 7.69 (d, *J* = 15.4 Hz, 1H, CH_arom._), 7.76–7.85 (m, 5H, CH_arom._), 8.16 (d, *J* = 8.7 Hz, 2H, CH_arom._); ^13 ^C NMR (100 MHz, DMSO-d_6_) *δ* 55.8, 64.7, 114.9, 115.0, 120.0, 129.0, 129.1, 129.4, 130.0, 131.1, 131.3, 135.9, 137.1, 143.9, 157.2, 161.8, 161.9, 176.2, 187.9; -ESI-MS (*m*/*z*): 463.3, [M-H]^−^; Anal. Calcd. for C_25_H_21_ClN_2_O_5_ (464.89): C, 64.59; H, 4.55; N, 6.03. Found: C, 64.47; H, 4.39; N, 6.08.

##### 2.1.1.4. *N’*-(2-{4-[(2*E*)-3-(4-Methoxyphenyl)prop-2-enoyl]phenoxy}acetoxy)-4-methoxybenzenecarboximidamide (4c)

Yield 89%; white solid; m.p.: 167–169 °C. IR (ATR) *ν*_max_ 3441, 3318 (NH_2_), 3063, 3004 (C–H aromatic), 2968, 2935, 2840 (C–H aliphatic), 1754 (C=O), 1653 (C=O), 1629 (C=N) cm^−1^. ^1^H NMR (400 MHz, DMSO-d_6_) *δ* 3.81 (s, 3H, OCH_3_), 3.83 (s, 3H, OCH_3_), 5.14 (s, 2H, CH_2_), 6.85 (s, 2H, NH_2_), 7.02 (d, *J* = 8.2 Hz, 4H, CH_arom._), 7.14 (d, *J* = 8.0 Hz, 2H, CH_arom._), 7.68–7.85 (m, 6H, CH_arom._), 8.16 (d, *J* = 8.0 Hz, 2H, CH_arom._); ^13^C NMR (100 MHz, DMSO-d_6_) *δ* 55.7, 55.8, 64.8 (exchangeable with dept-135), 114.2, 114.9, 115.0, 120.1, 123.8, 127.9, 128.8, 131.1, 131.2, 131.8, 143.7, 157.7, 161.6, 161.7, 162.0, 167.2, 187.9; -ESI-MS (*m*/*z*): 459.2, [M-H]^−^; Anal. Calcd. for C_26_H_24_N_2_O_6_ (460.47): C, 67.82; H, 5.25; N, 6.08. Found: C, 67.70; H, 5.46; N, 6.14.

##### 2.1.1.5. *N’*-(2-{4-[(2*E*)-3-(4-Methoxyphenyl)prop-2-enoyl]phenoxy}acetoxy)-3,4-dimethoxybenzenecarboximidamide (4d)

Yield 88%; white solid; m.p.: 150–152 °C. IR (ATR) *ν*_max_ 3451, 3329 (NH_2_), 3072 (C–H aromatic), 2978, 2936, 2839 (C–H aliphatic), 1755 (C=O), 1654 (C=O), 1626 (C=N) cm^−1^. ^1^H NMR (400 MHz, DMSO-d_6_) *δ* 3.78 (s, 9H, 3OCH_3_), 5.08 (s, 2H, CH_2_), 6.80 (s, 2H, NH_2_), 6.98–7.01 (m, 3H, CH_arom._), 7.12 (d, *J* = 8.3 Hz, 2H, CH_arom._), 7.26 (s, 1H, CH_arom._), 7.31 (d, *J* = 8.4 Hz, 1H, CH_arom._), 7.67 (d, *J* = 6.0 Hz, 2H, CH_arom._), 7.76 (d, *J* = 8.2 Hz, 2H, CH_arom._), 8.10 (d, *J* = 8.3 Hz, 2H, CH_arom._); ^13^C NMR (100 MHz, DMSO-d_6_) *δ* 55.8, 56.01, 56.03, 64.7, 110.5, 111.7, 114.9, 115.0, 119.9, 120.2, 123.8, 127.9, 131.18, 131.22, 131.7, 143.8, 148.8, 151.3, 157.9, 161.7, 162.0, 167.3, 187.8, 191.8; -ESI-MS (*m*/*z*): 489.5, [M-H]^−^; Anal. Calcd. for C_27_H_26_N_2_O_7_ (490.50): C, 66.11; H, 5.34; N, 5.71. Found: C, 66.01; H, 5.55; N, 5.62.

##### 2.1.1.6. *N’*-(2-{4-[(2*E*)-3-(4-Methoxyphenyl)prop-2-enoyl]phenoxy}acetoxy)-4-nitrobenzenecarboximidamide (4e)

Yield 84%; pale yellow solid; m.p.: 184–186 °C. IR (ATR) *ν*_max_ 3508, 3374 (NH_2_), 3074, 3019 (C–H aromatic), 2979, 2935, 2840 (C–H aliphatic), 1774 (C=O), 1655 (C=O), 1627 (C=N) cm^−1^. ^1^H NMR (400 MHz, DMSO-d_6_) *δ* 3.83 (s, 3H, OCH_3_), 5.18 (s, 2H, CH_2_), 7.02 (d, *J* = 8.6 Hz, 2H, CH_arom._), 7.15 (d, *J* = 8.7 Hz, 2H, CH_arom._), 7.25 (s, 2H, NH_2_), 7.67–7.84 (m, 4H, CH_arom._), 8.03 (d, *J* = 8.7 Hz, 2H, CH_arom._), 8.16 (d, *J* = 8.8 Hz, 2H, CH_arom._), 8.32 (d, *J* = 8.8 Hz, 2H, CH_arom._); ^13^C NMR (100 MHz, DMSO-d_6_) *δ* 55.8, 64.7, 114.9, 115.0, 120.1, 124.0, 127.9, 128.8, 131.1, 131.2, 131.9, 137.8, 143.7, 149.3, 156.6, 161.7, 161.9, 167.1, 187.9; -ESI-MS (*m*/*z*): 474.2, [M-H]^−^; Anal. Calcd. for C_25_H_21_N_3_O_7_ (475.45): C, 63.15; H, 4.45; N, 8.84. Found: C, 63.42; H, 4.23; N, 8.78.

##### 2.1.1.7. *N’*-(2-{4-[(2*E*)-3-(4-Methoxyphenyl)prop-2-enoyl]phenoxy}acetoxy) naphthalene-2-carboximidamide (4f)

Yield 85%; beige solid; m.p.: 176–178 °C. IR (ATR) *ν*_max_ 3437, 3319 (NH_2_), 3056 (C–H aromatic), 2995, 2966, 2933, 2834 (C–H aliphatic), 1754 (C=O), 1652 (C=O), 1636 (C=N) cm^−1^. ^1^H NMR (400 MHz, DMSO-d_6_) *δ* 3.83 (s, 3H, OCH_3_), 5.20 (s, 2H, CH_2_), 7.02 (d, *J* = 8.1 Hz, 2H, CH_arom._), 7.11 (s, 2H, NH_2_), 7.17 (d, *J* = 8.3 Hz, 2H, CH_arom._), 7.59–7.61 (m, 2H, CH_arom._), 7.69–7.88 (m, 5H, CH_arom._), 7.97–8.03 (m, 3H, CH_arom._), 8.18 (d, *J* = 8.3 Hz, 2H, CH_arom._), 8.37 (s, 1H, CH_arom._); ^13^C NMR (100 MHz, DMSO-d_6_) *δ* 55.8, 64.8, 114.9, 115.1, 120.1, 124.4, 127.1, 127.2, 127.8, 127.9, 128.1, 128.4, 129.0, 129.1, 131.1, 131.2, 131.9, 132.8, 134.4, 143.7, 158.0, 161.7, 162.0, 167.2, 187.9; -ESI-MS (*m*/*z*): 479.3, [M-H]^−^; Anal. Calcd. for C_29_H_24_N_2_O_5_ (480.51): C, 72.49; H, 5.03; N, 5.83. Found: C, 72.58; H, 4.89; N, 5.78.

##### 2.1.1.8. *N’*-(2-{4-[(1*E*)-3-(4-Methoxyphenyl)-3-oxoprop-1-en-1-yl] phenoxy}acetoxy) benzenecarboximidamide (6a)

Yield 82%; white solid; m.p.: 172–173 °C. IR (ATR) *ν*_max_ 3399, 3302 (NH_2_), 3045 (C–H aromatic), 2984, 2896 (C–H aliphatic), 1745 (C=O), 1666 (C=O), 1626 (C=N) cm^−1^. ^1^H NMR (400 MHz, DMSO-d_6_) *δ* 3.81 (s, 3H, OCH_3_), 5.04 (s, 2H, CH_2_), 7.07 (s, 2H, NH_2_), 7.20–7.22 (d, *J* = 7.9 Hz, 2H, CH_arom._), 7.29–7.72 (m, 6H, CH_arom._), 7.81–7.87 (m, 5H, CH_arom._), 8.01–8.02 (d, *J* = 7.8 Hz, 2H, CH_arom._); ^13^C NMR (100 MHz, DMSO-d_6_) *δ* 55.2, 63.8, 113.9, 115.2, 121.2, 125.2, 125.9, 127.9, 128.8, 130.2, 131.1, 131.6, 131.9, 144.6, 158.5, 161.3, 163.0, 167.1, 179.8; -ESI-MS (*m*/*z*): 429.3, [M-H]^−^; Anal. Calcd. for C_25_H_22_N_2_O_5_ (430.45): C, 69.76; H, 5.15; N, 6.51. Found: C, 69.65; H, 5.00; N, 6.41.

##### 2.1.1.9. *N’*-(2-{4-[(1*E*)-3-(4-Methoxyphenyl)-3-oxoprop-1-en-1-yl] phenoxy}acetoxy) -4-chlorobenzenecarboximidamide (6b)

Yield 85%; white solid; m.p.: 192–193 °C. IR (ATR) *ν*_max_ 3406, 3343 (NH_2_), 3062 (C–H aromatic), 2983, 2875 (C–H aliphatic), 1758 (C=O), 1663 (C=O), 1629 (C=N) cm^−1^. ^1^H NMR (400 MHz, DMSO-d_6_) *δ* 3.82 (s, 3H, OCH_3_), 4.98 (s, 2H, CH_2_), 7.00–7.29 (m, 6H, NH_2_ + CH_arom._), 7.49–7.51 (d, *J* = 8.1 Hz, 2H, CH_arom._), 7.70–7.91 (m, 6H, CH_arom._), 8.21–8.23 (d, *J* = 8.4 Hz, 2H, CH_arom._); ^13^C NMR (100 MHz, DMSO-d_6_) *δ* 55.5, 64.7, 113.5, 114.8, 115.7, 118.6, 122.3, 127.4, 130.4, 131.5, 132.7, 133.4, 139.8, 147.2, 147.8, 161.3, 162.9, 169.3, 179.9; -ESI-MS (*m*/*z*): 463.4, [M-H]^−^; Anal. Calcd. for C_25_H_21_ClN_2_O_5_ (464.89): C, 64.59; H, 4.55; N, 6.03. Found: C, 64.69; H, 4.27; N, 6.11.

##### 2.1.1.10. *N’*-(2-{4-[(1*E*)-3-(4-Methoxyphenyl)-3-oxoprop-1-en-1-yl] phenoxy} acetoxy)-4-methoxybenzenecarboximidamide (6c)

Yield 83%; white solid; m.p.: 186–187 °C. IR (ATR) *ν*_max_ 3422, 3365 (NH_2_), 3055, 3010 (C–H aromatic), 2989, 2873 (C–H aliphatic), 1751 (C=O), 1659 (C=O), 1631 (C=N) cm^−1^. ^1^H NMR (400 MHz, DMSO-d_6_) *δ* 3.83 (s, 3H, OCH_3_), 3.85 (s, 3H, OCH_3_), 5.06 (s, 2H, CH_2_), 6.97–7.07 (m, 6H, NH_2_ + CH_arom._), 7.12–7.14 (d, *J* = 8.3 Hz, 2H, CH_arom._), 7.27–7.29 (d, *J* = 8.0 Hz, 2H, CH_arom._), 7.92–8.05 (m, 6H, CH_arom._); ^13^C NMR (100 MHz, DMSO-d_6_) *δ* 55.8, 55.9, 64.5, 113.5, 114.8, 115.2, 117.1, 124.2, 127.2, 128.8, 131.0, 131.4, 131.9, 153.1, 158.4, 159.4, 161.4, 162.7, 169.9, 180.1; -ESI-MS (*m*/*z*): 459.4 [M-H]^−^; Anal. Calcd. for C_26_H_24_N_2_O_6_ (460.47): C, 67.82; H, 5.25; N, 6.08. Found: C, 67.62; H, 5.08; N, 6.02.

##### 2.1.1.11. *N’*-(2-{4-[(1*E*)-3-(4-Methoxyphenyl)-3-oxoprop-2-en-1-yl] phenoxy} acetoxy) -3,4-dimethoxybenzenecarboximidamide (6d)

Yield 82%; white solid; m.p.: 172–174 °C. IR (ATR) *ν*_max_ 3462, 3378 (NH_2_), 3058 (C–H aromatic), 2987, 2947, 2845 (C–H aliphatic), 1744 (C=O), 1652 (C=O), 1627 (C=N) cm^−1^. ^1^H NMR (400 MHz, DMSO-d_6_) *δ* 3.85 (s, 3H, OCH_3_), 3.89 (s, 3H, OCH_3_), 3.92 (s, 3H, OCH_3_), 5.15 (s, 2H, CH_2_), 6.96 (s, 2H, NH_2_), 7.08 (t, *J* = 8 Hz, 3H, CH_arom_), 7.13 (t, *J* = 8 Hz, 3H, CH_arom._), 7.34 (s, 1H, CH_arom._), 7.39 (d, *J* = 8 Hz, 1H, CH_arom_), 7.69–7.79 (m, 1H, CH_arom._), 7.93 (d, *J =* 8 Hz, 2H, CH_arom._), 8.22 (d, *J* = 8 Hz, 2H, CH = CH); ^13^C NMR (100 MHz, DMSO-d_6_) *δ* 56.0, 56.1, 64.6, 110.5, 111.7, 114.5, 115.4, 116.7, 120.1, 120.3, 123.8, 128.6, 131.1, 131.3, 132.2, 135.6, 137.3, 143.4, 148.8, 151.2, 157.8, 160.1, 163.6, 167.4, 187.7; -ESI-MS (*m*/*z*): 489.5, [M-H]^−^; Anal. Calcd. for C_27_H_26_N_2_O_7_ (490.50): C, 66.11; H, 5.34; N, 5.71. Found: C, 66.23; H, 5.27; N, 5.64.

##### 2.1.1.12. *N’*-(2-{4-[(1*E*)-3-(4-Methoxyphenyl)-3-oxoprop-2-en-1-yl] phenoxy} acetoxy)-4-nitrobenzenecarboximidamide (6e)

Yield 89%; pale yellow solid; m.p.: 193–194 °C. IR (ATR) *ν*_max_ 3452, 3364 (NH_2_), 3075, 3022 (C–H aromatic), 2989, 2932, 2844 (C–H aliphatic), 1769 (C=O), 1660 (C=O), 1628 (C=N) cm^−1^. ^1^H NMR (400 MHz, DMSO-d_6_) *δ* 3.84 (s, 3H, OCH_3_), 5.12 (s, 2H, CH_2_), 6.99–7.01 (d, *J* = 8.3 Hz, 2H, CH_arom._), 7.12–7.20 (m, 4H, NH_2_ + CH_arom._), 7.71–7.80 (m, 4H, CH_arom._), 8.07–8.09 (d, *J* = 8.4 Hz, 2H, CH_arom._), 8.13–8.15 (d, *J* = 8.7 Hz, 2H, CH_arom._), 8.29–8.31 (d, *J* = 8.7 Hz, 2H, CH_arom._); ^13 ^C NMR (100 MHz, DMSO-d_6_) *δ* 55.6, 64.8, 114.9, 115.4, 123.1, 125.5, 128.3, 128.9, 131.1, 131.6, 132.4, 135.3, 143.7, 148.6, 156.6, 161.8, 163.2, 167.4, 178.7; -ESI-MS (*m*/*z*): 474.4, [M-H]^−^; Anal. Calcd. for C_25_H_21_N_3_O_7_ (475.45): C, 63.15; H, 4.45; N, 8.84. Found: C, 63.36; H, 4.30; N, 8.90.

##### 2.1.1.13. *N’*-(2-{4-[(1*E*)-3-(4-Methoxyphenyl)-3-oxoprop-2-en-1-yl] phenoxyacetoxy)naphthalene-2-carboximidamide (6f)

Yield 86%; beige solid; m.p.: 178–179 °C. IR (ATR) *ν*_max_ 3427, 3353 (NH_2_), 3057 (C–H aromatic), 2992, 2952, 2854 (C–H aliphatic), 1752 (C=O), 1653 (C=O), 1633 (C=N) cm^−1^. ^1^H NMR (400 MHz, DMSO-d_6_) *δ* 3.80 (s, 3H, OCH_3_), 5.15 (s, 2H, CH_2_), 7.05–7.12 (m, 4H, NH_2_ + CH_arom._), 7.18–7.20 (d, *J* = 8.1 Hz, 2H, CH_arom._), 7.60–7.76 (m, 7H, CH_arom._), 7.94–8.00 (m, 3H, CH_arom._), 8.16–8.18 (d, *J* = 8.5 Hz, 2H, CH_arom._), 8.39 (s, 1H, CH_arom._); ^13^C NMR (100 MHz, DMSO-d_6_) *δ* 55.8, 64.9, 114.2, 114.9, 118.3, 125.4, 126.7, 126.9,127.5, 127.6, 128.3, 128.5, 129.0, 129.6, 131.1, 131.3, 131.9, 132.8, 134.6, 143.3, 149.6, 160.0, 162.0, 167.6, 180.1; -ESI-MS (*m*/*z*): 479.2, [M-H]^−^; Anal. Calcd. for C_29_H_24_N_2_O_5_ (480.51): C, 72.49; H, 5.03; N, 5.83. Found: C, 72.64; H, 4.82; N, 6.01.

### 2.2. Biology

#### 2.2.1. Cytotoxicity assay

MTT assay was used to assess the cell viability of RAW 264.7 cells cultured in RPMI-1640 complete media. The cells were treated with the investigated compounds two hours prior to 1 µg/ml of lipopolysaccharide (LPS) stimulation for 18 h. Afterwards, 5 µl of MTT solution were added and incubated for further 4 h. Finally, 150 ul of dimethyl sulfoxide (DMSO) were added and the optical density was assessed at 570 nm using an ELISA plate reader^46^.

#### 2.2.2. Griess assay for NO release determination

Equal volumes of Griess reagent and the supernatant, obtained from treated RAW264.7 cells with the compounds 2 h before LPS induction, were mixed for 10 min at room temperature in the dark. The absorbance was measured at 540 nm using ELISA plate reader and the nitrite concentration was calculated from sodium nitrite standard curve. The percentage inhibition (%) is calculated as absorbance at 540 nm of (LPS-compounds)/absorbance at 540 nm of (LPS-control) ×100^47^^,^^48^.

#### 2.2.3. Determination of iNOS enzymatic activity

Two hours after treatment with 2–50 µM of **4c, 4d, 6a, 6c, 6d, 6e,** indomethacin, and 1 µg/ml of LPS at 37 °C, the culture media was replaced by 100 µl of NOS assay buffer (1×). Then, the NOS assay reaction solution (100 µl/well) was added and incubated for an extra 2 h at 37 °C. The fluorescence with excitation wavelength at 485 nm and emission wavelength at 528 nm was measured using a fluorescent microplate reader^49^.

#### 2.2.4. Assessment of prostaglandin E2 concentration

After seeding and incubation of RAW 264.7 cells for 24 h, then the cells were treated with the compounds (**4c**, **4d**, **6a**, **6c**, **6d** and **6e**) at different concentrations and with LPS (1 µg/ml) for another 24 h. The concentration of prostaglandin E2 (PGE2) was measured in the culture media using ELISA kit (R&D Systems, Minneapolis, MN)^50^.

#### 2.2.5. *In vitro* cyclooxygenase (COX) inhibition assay

The colorimetric COX-1/COX-2 inhibition assay kit (kit catalogue number 560101, Cayman Chemical, Ann Arbour, MI) was used following the manufacturer’s instructions to test the ability of the test compounds and celecoxib to inhibit COX-1/COX-2 isozymes^3^^,^^12^.

#### 2.2.6. *In vitro* 5-lipoxygenase (LOX) inhibition assay

The 5-LOX inhibition assay kit (kit catalogue number 760700, Cayman Chemical, Ann Arbour, MI) was used following the manufacturer’s instructions to test the ability of the test compounds and NDGA to inhibit 5-LOX enzyme^3^^,^^12^.

#### 2.2.7. Carrageenan-induced paw edoema

The anti-inflammatory activity of the inspiring active compounds *in vitro* (**4c, 4d, 6a, 6c, 6d** and **6e**) were further estimated *in vivo* using the carrageenan-induced paw edoema test. The paw edoema was induced by a single injection of 1% w/v carrageenan (1 g dissolved in 100 ml saline) into the left hind paw. The paw thickness was assessed using Vernier calliper after carrageenan injection for 1,2, 3 and 4 h. Wistar albino rats (males weighing 120–140 g, six rats per group) were grouped as follows: Group 1 (Control) rats were received the vehicle. Groups 2 and 3 (standard-treated) rats were given 50 mg/kg oral dose of celecoxib or indomethacin as standard anti-inflammatory drugs. Groups 4–9 (compound-treated) each group of rats has orally administered one compound (**4c, 4d, 6a, 6c, 6d** or **6e**) at a dose of 50 mg/kg 1 h before paw edoema induction. The percentage inhibition of edoema thickness was calculated at each time interval in comparison to the control group^51^.

#### 2.2.8. Acute oral toxicity experiment

The compounds (**4c**, **4d**, **6a**, **6c**, **6d** and **6e**) were examined for the possible acute oral toxicity after their oral administration to male mice at doses of 100, 200, 300, 400 and 500 mg/kg, respectively. Twenty-four hours later, mice were observed for any signs of toxicity, and dead mice were recorded. The median lethal dose (LD_50_) was calculated according to Litchfield and Wilcoxon method^52^.

### 2.3. Docking methodology

For molecular docking analysis, Discovery Studio 2.5 software (Accelrys Inc., San Diego, CA) was used. The crystal structures of iNOS protein (PDB code: 1r35) was retrieved from protein data bank^1^^,^^10^^,^^53–56^. See Supplementary file.

### 2.4. *In silico* prediction of physicochemical properties and pharmacokinetic profile

For Lipinski’s rule (rule of five) and molecular property prediction, the free accesses to website (https://www.molsoft.com/servers.html) was used. Also, for Pre-ADMET estimation, the free access of website (https://preadmet.bmdrc.kr/) was utilised for estimation.

Approval and the corresponding ethical approval code.

## 3. Results and discussion

### 3.1. Chemistry

**Scheme 1.**
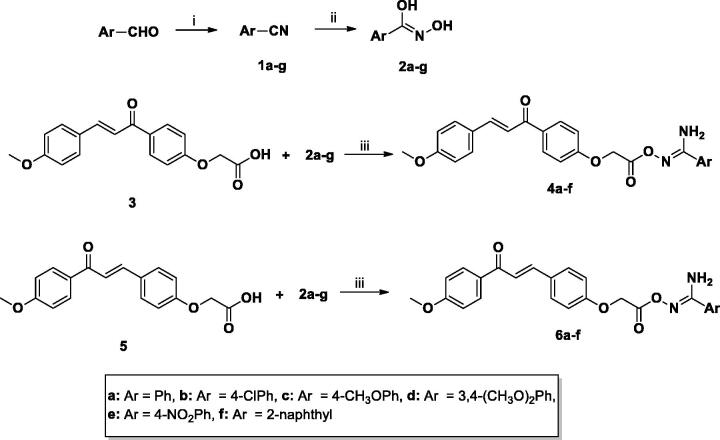
Synthesis of the target compounds **4a–f** and **6a–f**. *Reagents and conditions:* (i) I_2_, NH_3_, THF, rt, 1–2 h; (ii) NH_2_OH.HCl, K_2_CO_3_, MeOH, reflux; 5–8 h; (iii) CDI, CH_3_CN, rt, 3 h.

The chemical synthetic approach of the target chalcone/aryl carboximidamides **4a–f** and **6a–f** is outlined in Scheme 1. The intermediate aryl cyanides **1a–f**, amidoximes **2a–f** and the key chalcones acid **3** and **5** were prepared according to the previously reported procedure^10^^,^^12^^,^^22^^,^^57^. Reacting the synthesised amidoximes **2a–f** with the carboxylic acid group of chalcone **3** and **5** using carbonyldiimidazole (**CDI**) in acetonitrile gave the chalcone/aryl carboximidamides **4a–f** and **6a–f**, respectively, in good yield. The structure of the newly synthesised chalcone/aryl carboximidamides **4a–f** and **6a–f** was characterised by IR, ^1^H NMR,^13^CNMR spectra and elemental analyses. The IR spectrum of **4b**, as an example, displayed characteristic absorption bands at 3379, 3508 (NH_2_); 1763 (C=O), 1654 (C=O_enone_) and 1621(C=N) cm^−1^. While its ^1^H NMR spectrum indicates two singlet signals at *δ* 3.83 and 5.14 ppm assigned to methoxy and methylene protons, respectively. The amidoxime NH_2_ appeared at 7.01–7.03 ppm and the aromatic protons appeared in their expected chemical shifts. In addition, a characteristic doublet signal at *δ* 7.67–7.71 ppm assigned to be one of olefin hydrogen with coupling constant 15.4 Hz, which confirm the E configuration. The ^13^C NMR spectra as well as elemental analyses results are consistent with the suggested structures (see Supporting Information).

The IR spectrum of **6d** as a typical example of this sets revealed characteristic bands at 3462, 3378 (NH_2_); 1744 (C=O), 1652 (C=O_enone_) and 1627 (C=N) cm^−1^. ^1^H NMR spectrum revealed two singlet signals at *δ* 3.81 and 3.87 three methoxy and methylene protons, and 5.14 ppm assigned to methylene protons. The aromatic protons appeared in their expected chemical shifts while the amidoxime NH_2_ appeared at 6.84–6.86 ppm. In addition, a characteristic doublet signal at *δ* 7.67–7.71 ppm assigned to be one of olefin hydrogen with coupling constant 15.4 Hz, which confirm E configuration. The ^13^C NMR data and elemental microanalyses data are consistent with the expected structures.

### 3.2. Biology

#### 3.2.1. Determination of the cytotoxicity

**Figure 2.**
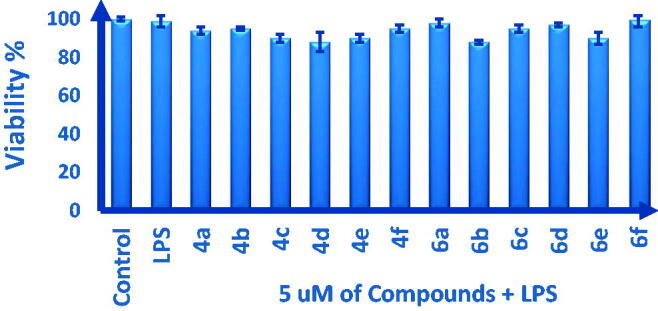
Cytotoxicity assessment of treatment with **4a–f** and **6a–f** compounds on RAW 264.7 cells, two hours before the induction with LPS. Cell viability was performed using MTT assay. Data are shown as mean ± SD (*n* = 3). *^,^** (*p* < 0.05) compared to untreated control cells and LPS-induced cells, respectively.

First, the cytotoxic effect of the synthesised target compounds **4a–f** and **6a–f** on cell growth and proliferation were evaluated. As illustrated in Figure 2, treatment of RAW 264.7 cells with 5 µM of compounds 2 h before induction with LPS did not show any noticeable cytotoxicity in comparison with untreated control cells as well as LPS-treated cells. Accordingly, other bioactivities were further assessed.

#### 3.2.2. Inhibitory activity of NO production

The important pro-inflammatory mediator, nitric oxide (NO), plays a vital role in the pathogenesis of several inflammatory diseases. Moreover, studies have demonstrated a positive link between the exaggerated concentration of NO and the severity of the disease serving NO as a potential biomarker in the evaluation of the inflammatory process. Therefore, NO inhibitors are essential therapies in the management of inflammatory disorders^58–60^.

Hence, we investigated the potential inhibitory effect of the two series on the over-production of NO from LPS-stimulated macrophages. As demonstrated in Table 1, compounds **4a–f** and **6a–f** at 5 µM showed more potent inhibition of NO production over the used standard drug, indomethacin (inhibitory rate= 25.5 ± 1.6%). Among the two series, compounds **4d** and **6d** were the most effective NO inhibitors with inhibition rates of 73.5 **±** 2.1 and 78.2 **±** 3.4%, respectively. On the contrary, compounds **4f** and **6f** were the least effective NO inhibitors displaying inhibitory rates of 31.2 **±** 2.2 and 29.4 **±** 2.5%, respectively. Compounds **4a, 4b, 4c, 4e, 6a, 6b, 6c** and **6e** displayed promising NO inhibition activity with inhibitory rates varying from 41.9% to 67.1%.

**Table 1.** The inhibition rates of NO release from RAW 264.7 cells after treatment with compounds **4a–f** and **6a–f**.

Compounds (5 µM)	NO Inhibition rates (%) ± SD	Compounds (5 µM)	NO Inhibition rates (%) ± SD
Indomethacin 25.5 ± 1.6			
4a	41.9 ± 1.4	6a	55.5 ± 2.4
4b	47.6 ± 3.6	6b	49.6 ± 2.3
4c	62.3 ± 1.9	6c	67.1 ± 2.4
4d	73.5 ± 2.1	6d	78.2 ± 3.4
4e	44.1 ± 2.8	6e	57.9 ± 1.3
4f	31.2 ± 2.2	6f	29.4 ± 2.5

#### 3.2.3. Inhibitory activity of iNOS and PGE2 production

NO is a core signalling mediator involved in the inflammation through iNOS up-regulation with the subsequent triggering of PGE2 induction and overstated inflammation^10^^,^^61^. Therefore, compounds (**4c**, **4d**, **6a**, **6c**, **6d** and **6e**) demonstrated an excellent inhibition rate of NO release from RAW 264.7 cells, were evaluated for potential inhibition on iNOS activity, NO, and PGE2 production.

As shown in Table 2, the treatment of LPS-induced RAW 264.7 cells with the selected six compounds resulted in a remarkable inhibition of iNOS activity (IC_50_ range from 1.91 to 7.15 µM) and NO (IC_50_ range = 4.36–15.80 µM) compared to indomethacin (IC_50_ 24.57 µM and 45.69 µM, respectively. Interestingly, the six compounds presented more inhibitory activity on the PGE2 production (IC_50_ range = 21.24–48.11 nM) in comparison with indomethacin (IC_50_ = 76.58 nM).

**Table 2.** NO, iNOS, PGE2 inhibitory activity (IC_50_) of compounds **4c**, **4d**, **6a**, **6c**, **6d** and **6e** and their cytotoxicity on RAW 264.7 cells.

Compound	NO IC_50_ (µM)	iNOS IC_50_ (µM)	PGE_2_ IC_50_ (nM)	Cytotoxicity IC_50_ (µM)	
				without LPS	with LPS
Indomethacin	45.69	24.57	76.58	>80	>80
**4c**	8.61	3.62	32.30	>80	>80
**4d**	5.73	2.22	24.72	>80	>80
**6a**	13.55	6.32	48.11	>80	>80
**6c**	5.92	2.45	27.51	>80	>80
**6d**	4.36	1.91	21.24	>80	>80
**6e**	15.80	7.15	45.14	>80	>80

Finally, the six active compounds were further evaluated for potential cytotoxicity using their inhibitory concentrations on NO, iNOS and PGE2 and the cell viability was assessed by MTT assay. Fortunately, the six compounds at the same inhibitory concentrations did not show notable cytotoxicity against RAW 264.7 cells with or without LPS presenting IC_50_ values >80 µM. This study proved that these compounds had promising inhibitory effects on LPS-stimulated inflammatory response without exerting cytotoxicity.

#### 3.2.4. *In vitro* COX-1/COX-2 inhibition assay

The six active compounds **4c**, **4d**, **6a**, **6c**, **6d** and **6e** were evaluated against both bovine COX-1 and COX-2 subtypes using enzyme immunoassay (EIA) kit using celecoxib as a reference drug. The IC_50_ values of the tested compounds along with their selectivity index (SI)^3^^,^^12^ are listed in Table 3. The obtained results revealed that compounds **4c**, **6a**, **6c** and **6e** are the most active COX-2 inhibitors with IC_50_ values of 3.279, 1.103, 8.263, 1.714 µM, respectively, compared to their COX-1 inhibitory activity (IC_50_ values of 19.88, 20.91, 64.41, 12.73 µM, respectively). Additionally, **6a** displayed the highest SI value of 19 compared to that of celecoxib (SI = 175.49). Unexpectedly, compounds **4d** and **6d** were proved to be more selective COX-1 inhibitor (IC_50_ = 19.88 and 17.82 µM, respectively) compared to their COX-2 inhibitory activity (IC_50_ = 23.4 and 102.7 µM, respectively).

**Table 3.** *In vitro* COX1, COX2 and 5LOX inhibitory activity (IC_50_) of compounds **4c**, **4d**, **6a**, **6c**, **6d** and **6e**.

Compound	COX 1 IC_50_ (µM)	COX 2 IC_50_ (µM)	SI	5LOX IC_50_
				(µM)
**4c**	19.88 ± 1.03	3.279 ± 0.16	6.06	8.136 ± 0.5
**4d**	5.824 ± 0.3	23.4 ± 1.13	0.25	16.47 ± 1
**6a**	20.91 ± 1.09	1.103 ± 0.05	19	6.126 ± 0.4
**6c**	64.41 ± 3.35	8.263 ± 0.4	7.79	3.186 ± 0.2
**6d**	17.82 ± 0.93	102.7 ± 4.97	0.17	9.877 ± 0.6
**6e**	12.73 ± 0.66	1.714 ± 0.08	7.43	54.15 ± 3.2
Celecoxib	35.8 ± 1.25	0.204 ± 0.06	175.49	89.4 ± 1.16
NDGA	na	na	na	2.96 ± 0.2

#### 3.2.5. *In vitro* 5-LOX inhibition assay

The six active compounds **4c**, **4d**, **6a**, **6c**, **6d** and **6e** were evaluated for their 5-LOX inhibitory activity. The IC_50_ values of the six compounds were determined and illustrated in Table 3. The results disclosed that compound **6c** exhibited good inhibitory activity towards 5-LOX enzyme (IC_50_ = 3.186 µM) compared to nordihydroguaiaretic acid (NDGA) (IC_50_ = 2.96 µM). Additionally, compounds **4c**, **4d**, **6a** and **6d** showed moderate ability to inhibit 5-LOX enzyme with IC_50_ values of 8.136, 16.4, 6.126 and 9.877 µM, respectively. Moreover, compound **6e** showed the weakest 5-LOX inhibitory activity (IC_50_ =54.15 µM).

#### 3.2.6. *In vivo* anti-inflammatory activity

Compounds that displayed promising *in vitro* bioactivities (**4c**, **4d**, **6a**, **6c**, **6d** and **6e**) were further assessed for *in vivo* anti-inflammatory activity using the carrageenan-induced rat paw edoema model. Celecoxib and indomethacin were used as standard anti-inflammatory drugs. As shown in Table 4, the paw thickness was measured at 1, 2, 3 and 4 h following oedema induction, and the results were demonstrated as percentage oedema inhibition.

**Table 4.** The anti-inflammatory activities of compounds **4a**, **4d**, **6a**, **6c**, **6d**, **6e**, indomethacin, and celecoxib against *in vivo* model of carrageenan-induced paw edoema.

% of edoema inhibition (% mean ± SEM)^a^				
Compound	1 h	2 h	3 h	4 h
Control	0.00	0.00	0.00	0.00
**4c**	11.21 ± 2.41	33.62 ± 2.69	57.71 ± 2.21	69.86 ± 3.51
**4d**	11.65 ± 2.26	34.78 ± 3.34	69.50 ± 1.49	54.21 ± 3.47
**6a**	14.36 ± 3.11	24.33 ± 2.26	45.83 ± 2.74	55.74 ± 2.59
**6c**	11.42 ± 1.75	28.44 ± 2.75	54.36 ± 2.34	62.21 ± 3.46
**6d**	13.25 ± 2.04	38.43 ± 3.62	65.31 ± 2.64	78.53 ± 3.28
**6e**	10.23 ± 3.17	19.34 ± 2.45	39.26 ± 18	33.21 ± 3.15
Indomethacin	28.03 ± 1.35	45.27 ± 2.48	63.71 ± 3.48	56.27 ± 2.14
Celecoxib	10.61 ± 2.59	25.43 ± 3.29	46.42 ± 2.87	12.32 ± 2.42

^a^Data are presented as means ± SEM (*n* = 6). The anti-inflammatory activity represented as percentage edoema inhibition was calculated as follows: the increase of paw thickness in the control group **–** (the increase of paw thickness in the treated group**/**the increase of paw thickness in the control group)**×**100.

Three hours after oedema induction, compounds **4c**, **4d**, **6a**, **6c** and **6d** displayed outstanding anti-inflammatory activity with oedema inhibition of 57.71%, 69.50%, 54.36% and 65.31%, respectively, compared to indomethacin (63.71%) and celecoxib (46.42%), while compound **6a** showed moderate anti-inflammatory activity with 45.83% decrease in oedema thickness. Consistent with the *in vitro* studies, compound **4e** demonstrated the least activity of oedema inhibition with 39.26%.

Strikingly, the anti-inflammatory activity of **4c**, **6c** and **6d** was remarkably increased four hours after carrageenan injection indicating a long-lasting inhibition activity of these compounds with 69.86%, 62.21% and 78.53% inhibition, respectively, compared to celecoxib (12.32%) and indomethacin (56.27%). Compound **6d** was the most potent one in this research showing nearly 7-fold and 1.5-fold more activity than the two used standard drugs celecoxib and indomethacin, respectively.

#### 3.2.7. Acute toxicity study

The most active compounds **4c, 4d, 6c** and **6d**, representing the maximum anti-inflammatory activity were administered p.o. to mice at doses of 100, 200, 300, 400 and 500 mg/kg. No treatment-associated toxic signs or deaths were detected or recorded at the tested concentrations suggesting the safety as well as the well-tolerability of these compounds.

## 4. Molecular docking study of iNOS (PDB ID: 1r35)

Discovery Studio 2.5 software was used to explore and better understand the potency and the ability of the evaluated compounds to fit nicely into the active site of iNOS protein, the most active compounds **4c**, **4d**, **6c** and **6d** were selected to inspect their binding with iNOS protein whose 3D crystal structure (PDB ID: 1r35) was downloaded from the Protein Data Bank^1^. The virtually docked compounds were built using ChemBioDraw Ultra 12.0 and finally to get the minimum lowest energy structure, the force fields were applied on compounds **4c**, **4d**, **6c** and **6d**. Then, the binding site sphere has been defined automatically by the software. The best obtained studied poses were chosen for docking using CDOCKER energy and were inspected in 3D and 2D styles^54–56^.

From the inspection of docking results, it is obvious that compounds **4a**, **4d**, **6c** and **6d** have the ability to nicely fit into iNOS (PDB ID: 1r35) catalytic binding pocket, demonstrating good uniformity between the *in vitro* iNOS screening and the in silico prediction.

**Figure 3.**
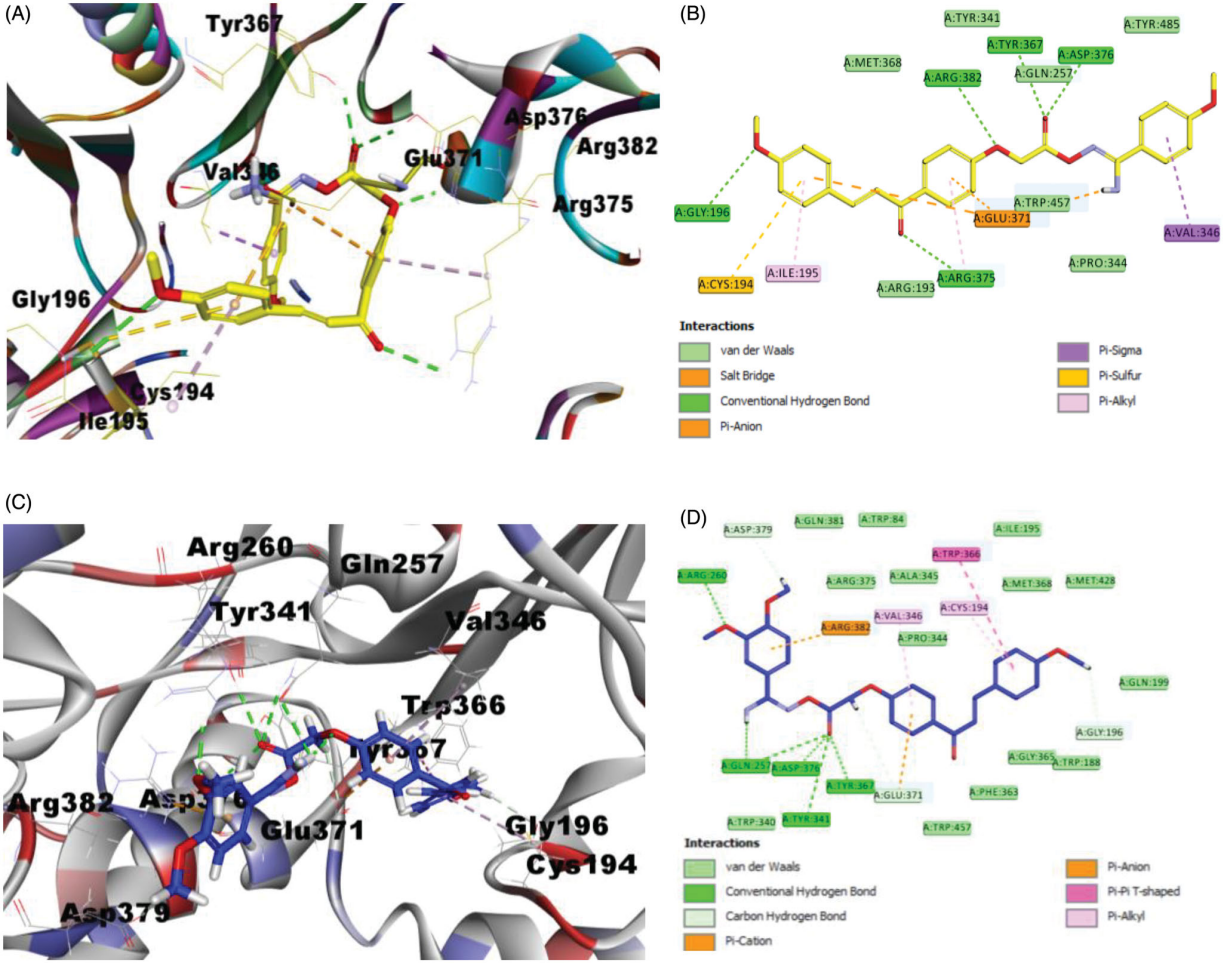
Binding mode of compounds **4c** and **4d** into iNOS pocket (PDB code: 1r35). (A) 3D structure of **4c** (yellow), (B) 2D structure of **4c** (yellow), (C) 3D structure of **4d** (blue) and (D) 2D structure of **4d** (blue).

The docking results of compound **4c** (CDOCKER energy = −37.0199 and CDOCKER interaction energy = −56.5283) (Figure 3(A,B)), revealed that it formed 5-H bonds; the oxygen of 4-methoxy formed one hydrogen bond with Gly196, the oxygen of the 1,3-propenone moiety engaged in one hydrogen bond with Arg375, the oxygen adjacent to methylene group incorporated in one hydrogen bond with Arg382 and finally the carbonyl oxygen of the carboximidamide group formed 2-H bond with Tyr367 and Asp376 amino acid residues. Additionally, **4c** showed many hydrophobic interactions such as Pi–Sigma interaction with Val346, Pi–sulphur interaction with Cys194, Salt Bridge and Pi–anion interactions with Glu371 and two Pi–alkyl interactions with Ile195 and Arg375 amino acid residues.

The found result of compound **4d** (Figure 3(C,D)) exhibited that **4d** has better binding scores than **4c** (CDOCKER energy = −40.445 and CDOCKER interaction energy = −66.5266). Moreover, **4d** formed 7-H bonds; the oxygen atom of one of the dimethoxy groups incorporated in one hydrogen bond with Arg260, the proton of the amino group of the carboximidamide formed one hydrogen bond with Gln275, while the carbonyl oxygen of the carboximidamide group engaged in 4-H bonds with Gln275, Tyr341, Tyr367 and Asp376 amino acid residues. Further, **4d** showed many hydrophobic interactions such as van der Waals, Carbon Hydrogen Bond, Pi–cation, Pi–anion, Pi–Pi T-shaped and Pi–alkyl interactions with Gly196, Glu371, Asp379, Arg382, Trp366, Cys194 and Val346 amino acid residues, respectively. These finding in agreement with the iNOS inhibition assay and explain the higher activity of compound **4d** more than **4c**.

**Figure 4.**
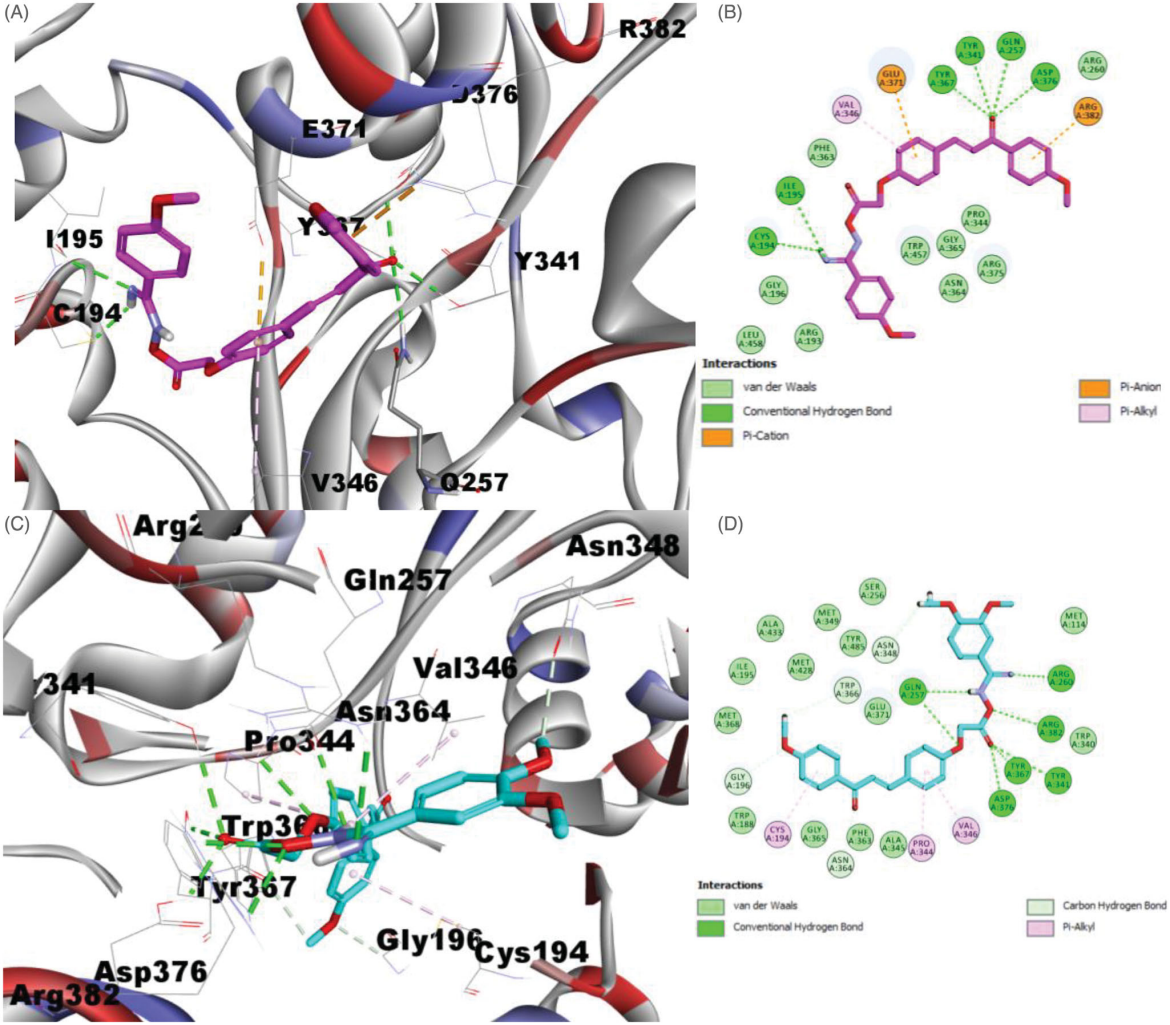
Binding mode of compounds **6c** and **6d** iNOS pocket (PDB code: 1r35). (A) 3D structure of **6c** (pink), (B) 2D structure of **6c** (pink), (C) 3D structure of **6d** (cyan) and (D) 2D structure of **6d** (cyan).

Concerning compound **6c** (Figure 4(A,B)), (CDOCKER energy = −43.0261 and CDOCKER interaction energy = −64.8146), involved into 6-H bonds; the carbonyl oxygen of the 1,3-propenone moiety formed 4-H bonds with Gln275, Tyr341, Tyr367 and Asp376 amino acid residues, the proton of the amino group of the carboximidamide engaged in 2-H bonds with Cys194 and Ile195 amino acid residues. Also, **6d** showed many hydrophobic interactions as van der Waals, Pi-Cation, Pi-Anion and Pi-Alkyl interactions with Glu371, Arg382, Val346 amino acid residues.

Finally, compound **6d** (Figure 4(C,D)), with −35.5756 CDOCKER energy and −67.008 CDOCKER interaction energy, incorporated in 8-H bonds; Arg260 formed one hydrogen bond with the nitrogen atom of the of the carboximidamide group, Glu257 engaged in 2-H bond with the proton of the amino group of the carboximidamide group and, the oxygen adjacent to methylene group, Arg382 incorporated in 2-H bond with the oxygen atom of the carboximidamide group, wherein Tyr341, Tyr367 and Asp376 each engaged in one hydrogen bond with carbonyl oxygen of the carboximidamide group.

From the inspection of the docking results, it could be concluded that, compounds **4c**, **4d**, **6d** and **6d**, particularly, **4d** and **6d** fit nicely into the pocket of iNOS protein and they are entitled to be used as future lead template for identifying more potent anti-inflammatory candidates.

## 5. *In silico* prediction of physicochemical properties and pharmacokinetic profile

### 5.1. Lipinski rule calculations and ADMET analysis

Prediction of the physicochemical characters, pharmacokinetics and toxicity is an important tool in drug discovery of biologically active agents^10^^,^^62^. Thus, the most active derivatives **4c**, **4d**, **6c** and **6d** were analysed for prediction of Lipinski’s Rule of Five^63^ and Veber’s standard^64^. Therefore, utilising the online application Pre-ADMET, theoretical calculations of the pharmacokinetic parameters as well as the theoretical agreement of the most potent compounds **4c**, **4d**, **6c** and **6d** to both Veber’s criteria and Lipinski’s rule of five were carried out^65^.

The obtained results as illustrated in Table 5 showed that compounds **4c**, **4d**, **6c** and **6d** are in full accordance to Lipinski’s rule without any violation. Moreover, all the tested compounds had TPSA values < 140 Å^2^ which used to calculate the percentage of oral absorption (%ABS) using the following equation: (%ABS = 109–(0.345 TPSA)^66^. The tested compounds **4c**, **4d**, **6c** and **6d** exhibited %ABS of 78.96, 76.30, 78.96 and 76.30, respectively.

**Table 5.** Calculated parameters of Veber's and Lipinski's rule of five for compounds **4c**, **4d**, **6c and 6d** and indomethacin.

Comp.	MW	Log P	HBD	HBA	nVs	TPSA	%ABS
Lipinski^a^	≤500	≤5	≤5	≤10	≤1	–	–
Veber^b^	–	–	–	–	–	≤140	–
**4c**	460.16	3.57	2	7	0	87.08 A^2^	78.96
**4d**	490.17	3.17	2	8	0	94.79 A^2^	76.30
**6c**	460.16	3.57	2	7	0	87.08 A^2^	78.96
**6d**	490.17	3.17	2	8	0	94.79 A^2^	76.30
Indomethacin	357.08	4.00	1	4	0	51.31 A^2^	91.30

^a^Reference values of Lipinski.

^b^Reference values of Veber; MW: molecular weight; LogP: lipophilicity (O/W); HBD: number of hydrogen bond donors; HBA: number of hydrogen bond acceptors; nVs: number of Lipinski rule violations; TPSA: topological polar surface area (TPSA) (Å2); %ABS: percentage of oral absorption.

Furthermore, the results as shown in Table 6 revealed that compounds **4c**, **4d**, **6c** and **6d** had intermediate cell permeability in the CaCO_2_ cell model they are expected to be excellently absorbed through the intestine with HIA values close to 1. Notably, all the tested compounds **4c**, **4d**, **6c** and **6d** were predicted to be non-toxic in Ames test and to be non-carcinogenic as shown in Table 6. From these results, it could be concluded that compound **4c**, **4d**, **6c** and **6d** had acceptable physicochemical properties and reasonable drug-likeness, hence, can be used as a promising drug candidate for development of new anti-inflammatory agents that act as dual iNOS/PGE2 inhibitors.

**Table 6.** Predicted ADMET data of compounds **4c**, **4d**, **6c**, **6d** and indomethacin.

Compound	HIA	CaCO_2_ value	Rule of five	MDDR-like rule	PPB	BBB	AMES toxicity	Carcinogenicity
**4c**	98.46	20.69	Suitable	Drug-like	90.97	0.212	Non-mutagen	Negative
**4d**	98.64	25.07	Suitable	Drug-like	86.73	0.202	Non-mutagen	Negative
**6c**	98.46	21.67	Suitable	Drug-like	90.97	0.161	Non-mutagen	Negative
**6d**	98.64	25.80	Suitable	Drug-like	86.73	0.164	Non-mutagen	Negative
Indomethacin	97.90	20.03	Suitable	Drug-like	89.55	0.027	Non-mutagen	Negative

HIA: human intestinal absorption (%); CaCO_2_: permeability through CaCO_2_ (human colorectal carcinoma) cells *in vitro*; PPB: plasma protein binding; BBB: blood brain barrier penetration.

## 6. Structure–activity relationship

**Figure 5.**
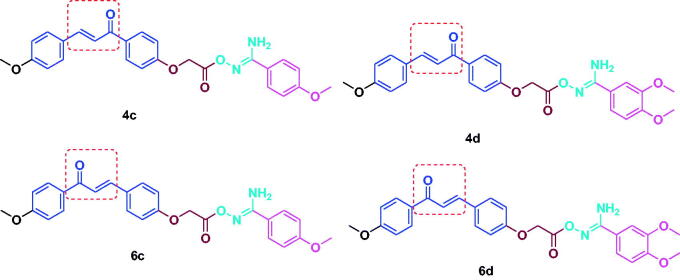
Chemical structures of the most active compounds **4c**, **4d**, **6c** and **6d**.

Study of the SAR showed that, in general, the chalcone/aryl carboximidamides **6a–f** proved to be more potent inhibitor of LPS induced NO production than chalcone/aryl carboximidamides **4a–f**. Ongoing throughout the results, it is obvious that the presence of donating groups leads to increase the suppression of LPS induced NO production. For instant, compounds **4d** and **6d**, with two methoxy group, exhibited the highest activity (73.5%, 78.2% inhibition, respectively). Removal of the 3-methoxy group of **4d** and **6d** (Figure 5) gave **4c** and **6c** with slight decrease in activity (62.3%, 67.1.8% inhibition, respectively). Removing the electron-donating two methoxy groups (as in **4a** and **6a**) or introducing electron-withdrawing such as Cl (**4b** and **6b**) or NO_2_ (**4e** and **6e**) resulted in decreased the NO release inhibition potency (41.9%, 55.5%, 47.6%, 49.6%, 44.1% and 57.9% inhibition, respectively). Replacing the phenyl group with the bulky naphthyl one yielded compounds **4f** and **6f** with dramatic decrease in activity (31.2%, 29.4% inhibition, respectively). The same SAR correlation could be applied for the obtained results of the *in vitro* iNOS, PGE2 inhibitory activity and the *in vivo* anti-inflammatory activity.

Regarding the COX inhibitory activity, it is obvious that the unsubstituted phenyl group is the optimal for COX2 inhibitory activity as in compound **6a** (1.103 ± 0.05 µM). introducing the strong electron withdrawing group NO2 retain the activity as in compound 6e (1.714 ± 0.08 µM) while introducing one donating group as methoxy results in a moderate decrease in the COX2 inhibitory activity as in compounds **4c** (3.279 ± 0.16 µM) and **6c** (8.263 ± 0.4 µM). On the other hand, introducing two methoxy groups leads to a dramatic decrease in the COX2 inhibitory activity as in compounds **4d** (23.4 ± 1.13 µM) and **6d** (102.7 ± 4.97 µM). Concerning the 5LOX inhibitory activity, compound **6c** with one methoxy group displayed the best inhibitory activity towards 5-LOX enzyme (IC_50_ = 3.186 µM). Converting the propanone moiety as in compound **4c** (8.136 ± 0.5 µM), removing the methoxy group as in compound **6a** (6.126 ± 0.4 µM), or adding another methoxy group as in compounds **4d** (16.47 ± 1 µM) and **6d** (9.877 ± 0.6 µM) result in a decrease in the 5LOX inhibitory activity. Finally, replacing the methoxy group with the strongly deactivating NO_2_ group as in compound **6e** (54.15 ± 3.2 µM) leads to a dramatic decrease in the 5LOX inhibitory activity. from all these results it is obvious that both the substituent and the propanone moiety affect the bioactivity of these compounds. Moreover, it is clear that propanone moiety in chalcones **6a–f** exhibited better bioactivity compared to its corresponding chalcones **4a–f**.

## 7. Conclusions

In summary, two series of chalcone linked to aryl carboximidamides **4a–f** and **6a–f** were designed, synthesised and evaluated for iNOS. PGE2 inhibitory activity as well as for their *in vivo* anti-inflammatory activity using carrageenan-induced paw oedema method. All the synthesised compounds (**4a–f** and **6a–f**) displayed significant iNOS inhibitory activity with IC_50_ values ranging from 2.31 to 9.48 µM) and NO (IC_50_ range of 6.5–13.01 µM) in comparison to indomethacin (IC_50_ 24.57 µM and 45.69 µ M, respectively), against LPS-induced RAW 264.7 cells. Compounds **4c**, **4d**, **6c** and **6d**, with one or two methoxy groups, proved to be the most potent LPS induced NO over-production inhibitors with 62.3%, 73.5%, 67.1% and 78.2%, respectively. Additionally, **4c**, **6a** and **6e** exhibited good COX2 inhibitory activity while **4c**, **6a** and **6c** showed the highest 5LOX inhibitory activity. Moreover, **4c**, **4d**, **6c** and **6d** exhibited significant *in vivo* anti-inflammatory activity with oedema inhibition of 69.86% (after 4 h), 69.5% (after 3 h), 62.21% (after 4 h) and 78.51% (after 4 h), respectively, compared to indomethacin (56.27%) (after 4 h) and celecoxib (12.32%) (after 4 h). Notably, compounds **4d** and **6d** were the most LPS-induced NO production, iNOS activity and PGE2 inhibitors. The docking study revealed that compounds **4c**, **4d**, **6c** and **6d** fit nicely into the iNOS protein pocket (PDB ID: 1r35) through the important amino acid residues and these results were in agreement with the obtained biological results of iNOS inhibition assay. Furthermore, the predicted parameters of Lipinski’s rule of five and ADMET analysis showed that **4c**, **4d**, **6c** and **6d** had acceptable physicochemical properties and good drug-likeness scores. Therefore, compound **4c**, **4d**, **6c** and **6d**, in particular **4d** and **6d**, could serve as promising lead as anti-inflammatory candidate which merit further structural optimisation for more precise SAR and more potent derivatives.

## Supplementary Material

Supplemental Material

## References

[1] Ma L, Pei H, Lei L, et al. Structural exploration, synthesis and pharmacological evaluation of novel 5-benzylidenethiazolidine-2,4-dione derivatives as iNOS inhibitors against inflammatory diseases. Eur J Med Chem 2015;92:178–90. 25555141 10.1016/j.ejmech.2014.12.036

[2] Medzhitov R. Inflammation 2010: new adventures of an old flame. Cell 2010;140:771–6. 20303867 10.1016/j.cell.2010.03.006

[3] Qandeel NA, El-Damasy AK, Sharawy MH, et al. El-Gohary NS. Synthesis, in vivo anti-inflammatory, COX-1/COX-2 and 5-LOX inhibitory activities of new 2,3,4-trisubstituted thiophene derivatives. Bioorg Chem 2020;102:103890. 32801081 10.1016/j.bioorg.2020.103890

[4] Abdulkhaleq LA, Assi MA, Abdullah R, et al. The crucial roles of inflammatory mediators in inflammation: a review. Vet World 2018;11:627–35. 29915501 10.14202/vetworld.2018.627-635 PMC5993766

[5] Ricciotti E, FitzGerald GA. Prostaglandins and inflammation. Arterioscler Thromb Vasc Biol 2011;31:986–1000. 21508345 10.1161/ATVBAHA.110.207449 PMC3081099

[6] Zelenay S, van der Veen Annemarthe G, Böttcher JP, et al. Cyclooxygenase-dependent tumor growth through evasion of immunity. Cell 2015;162:1257–70. 26343581 10.1016/j.cell.2015.08.015 PMC4597191

[7] Gomez I, Foudi N, Longrois D, Norel X. The role of prostaglandin E2 in human vascular inflammation. Prostaglandins Leukot Essent Fatty Acids 2013;89:55–63. 23756023 10.1016/j.plefa.2013.04.004

[8] Collot V, Sopkova-de Oliveira Santos J, Schumann-Bard P, et al. Synthesis, pharmacological study and modeling of 7-methoxyindazole and related substituted indazoles as neuronal nitric oxide synthase inhibitors. J Enzyme Inhib Med Chem 2003;18:195–9. 12943204 10.1080/1475636032000069864

[9] Huang H, Martásek P, Roman LJ, Silverman RB. Synthesis and evaluation of dipeptide amides containing N omega-nitroarginine and D-2,4-diaminobutyric acids as inhibitors of neuronal nitric oxide synthase. J Enzyme Inhib 2001;16:233–9. 11697043 10.1080/14756360109162371

[10] Mohamed MFA, Marzouk AA, Nafady A, et al. Design, synthesis and molecular modeling of novel aryl carboximidamides and 3-aryl-1,2,4-oxadiazoles derived from indomethacin as potent anti-inflammatory iNOS/PGE2 inhibitors. Bioorg Chem 2020;105:104439. 33161252 10.1016/j.bioorg.2020.104439

[11] Heemskerk S, Masereeuw R, Russel FGM, Pickkers P. Selective iNOS inhibition for the treatment of sepsis-induced acute kidney injury. Nat Rev Nephrol 2009;5:629–40. 19786992 10.1038/nrneph.2009.155

[12] Youssif BGM, Mohamed MFA, Al-Sanea MM, et al. Novel aryl carboximidamide and 3-aryl-1,2,4-oxadiazole analogues of naproxen as dual selective COX-2/15-LOX inhibitors: design, synthesis and docking studies. Bioorg Chem 2019;85:577–84. 30878890 10.1016/j.bioorg.2019.02.043

[13] Hayashi S, Ueno N, Murase A, Takada J. Design, synthesis and structure-activity relationship studies of novel and diverse cyclooxygenase-2 inhibitors as anti-inflammatory drugs. J Enzyme Inhib Med Chem 2014;29:846–67. 24517373 10.3109/14756366.2013.864650

[14] Abdellatif KR, Elsaady MT, Abdel-Aziz SA, Abusabaa AH. Synthesis, cyclooxygenase inhibition and anti-inflammatory evaluation of new 1,3,5-triaryl-4,5-dihydro-1H-pyrazole derivatives possessing methanesulphonyl pharmacophore. J Enzyme Inhib Med Chem 2016;31:1545–55. 27072288 10.3109/14756366.2016.1158168

[15] El-Subbagh HI, Hassan GS, El-Messery SM, et al. Nonclassical antifolates, part 5. Benzodiazepine analogs as a new class of DHFR inhibitors: synthesis, antitumor testing and molecular modeling study. Eur J Med Chem 2014;74:234–45. 24469112 10.1016/j.ejmech.2014.01.004

[16] Lin Y-M, Zhou Y, Flavin MT, et al. Chalcones and flavonoids as anti-tuberculosis agents. Bioorg Med Chem 2002;10:2795–802. 12057669 10.1016/s0968-0896(02)00094-9

[17] Bhat BA, Dhar KL, Puri SC, et al. Synthesis and biological evaluation of chalcones and their derived pyrazoles as potential cytotoxic agents. Bioorg Med Chem Lett 2005;15:3177–80. 15893928 10.1016/j.bmcl.2005.03.121

[18] Lévai A. Synthesis of chlorinated 3,5-diaryl-2-pyrazolines by the reaction of chlorochalcones with hydrazines. Arkivok 2005;2005:344–52.

[19] Azarifar D, Ghasemnejad H. Microwave-assisted synthesis of some 3,5-arylated 2-pyrazolines. Molecules 2003;8:642–8.

[20] Varga L, Nagy T, Kövesdi I, et al. Solution-phase parallel synthesis of 4, 6-diaryl-pyrimidine-2-ylamines and 2-amino-5, 5-disubstituted-3, 5-dihydro-imidazol-4-ones via a rearrangement. Tetrahedron 2003;59:655–62.

[21] Zhuang C, Zhang W, Sheng C, et al. Chalcone: a privileged structure in medicinal chemistry. Chem Rev 2017;117:7762–810. 28488435 10.1021/acs.chemrev.7b00020 PMC6131713

[22] Mohamed MF, Shaykoon MSA, Abdelrahman MH, et al. Design, synthesis, docking studies and biological evaluation of novel chalcone derivatives as potential histone deacetylase inhibitors. Bioorg Chem 2017;72:32–41. 28346873 10.1016/j.bioorg.2017.03.005

[23] Abou-Zied HA, Youssif BG, Mohamed MF, et al. EGFR inhibitors and apoptotic inducers: design, synthesis, anticancer activity and docking studies of novel xanthine derivatives carrying chalcone moiety as hybrid molecules. Bioorg Chem 2019;89:102997. 31136902 10.1016/j.bioorg.2019.102997

[24] Mohamed MF, Abuo-Rahma G-DA. Molecular targets and anticancer activity of quinoline–chalcone hybrids: literature review. RSC Adv 2020;10:31139–55. 10.1039/d0ra05594h PMC9056499 35520674

[25] Kumar H, Devaraji V, Joshi R, et al. Antihypertensive activity of a quinoline appended chalcone derivative and its site specific binding interaction with a relevant target carrier protein. RSC Adv 2015;5:65496–513.

[26] Liu M, Wilairat P, Croft SL, et al. Structure–activity relationships of antileishmanial and antimalarial chalcones. Bioorg Med Chem 2003;11:2729–38. 12788347 10.1016/s0968-0896(03)00233-5

[27] Narender T, Tanvir K, Rao MS, et al. Prenylated chalcones isolated from Crotalaria genus inhibits in vitro growth of the human malaria parasite Plasmodium falciparum. Bioorg Med Chem Lett 2005;15:2453–5. 15929201 10.1016/j.bmcl.2005.03.081

[28] Arty IS, Timmerman H, Samhoedi M, van der Goot H. Synthesis of benzylideneacetophenones and their inhibition of lipid peroxidation. Eur J Med Chem 2000;35:449–57. 10858605 10.1016/s0223-5234(00)00137-9

[29] Mukherjee S, Kumar V, Prasad AK, et al. Synthetic and biological activity evaluation studies on novel 1,3-diarylpropenones. Bioorg Med Chem 2001;9:337–45. 11249126 10.1016/s0968-0896(00)00249-2

[30] Viana G, Bandeira M, Matos F. Analgesic and antiinflammatory effects of chalcones isolated from *Myracrodruon urundeuva* Allemão. Phytomedicine 2003;10:189–95. 12725575 10.1078/094471103321659924

[31] Lee SH, Sohn DH, Jin XY, et al. 2′, 4′, 6′-Tris (methoxymethoxy) chalcone protects against trinitrobenzene sulfonic acid-induced colitis and blocks tumor necrosis factor-α-induced intestinal epithelial inflammation via heme oxygenase 1-dependent and independent pathways. Biochem Pharmacol 2007;74:870–80. 17678632 10.1016/j.bcp.2007.06.034

[32] Yadav P, Lal K, Kumar L, et al. Synthesis, crystal structure and antimicrobial potential of some fluorinated chalcone-1,2,3-triazole conjugates. Eur J Med Chem 2018;155:263–74. 29890388 10.1016/j.ejmech.2018.05.055

[33] Nowakowska Z. A review of anti-infective and anti-inflammatory chalcones. Eur J Med Chem 2007;42:125–37. 17112640 10.1016/j.ejmech.2006.09.019

[34] Go M, Wu X, Liu X. Chalcones: an update on cytotoxic and chemoprotective properties. Curr Med Chem 2005;12:483–99. 10.2174/0929867053363153 15720256

[35] Sashidhara KV, Avula SR, Mishra V, et al. Identification of quinoline-chalcone hybrids as potential antiulcer agents. Eur J Med Chem 2015;89:638–53. 25462272 10.1016/j.ejmech.2014.10.068

[36] Li R, Chen X, Gong B, et al. Structure-based design of parasitic protease inhibitors. Bioorg Med Chem 1996;4:1421–7. 8894100 10.1016/0968-0896(96)00136-8

[37] Trivedi JC, Bariwal JB, Upadhyay KD, et al. Improved and rapid synthesis of new coumarinyl chalcone derivatives and their antiviral activity. Tetrahedron Lett 2007;48:8472–4.

[38] Sivakumar PM, Babu SKG, Mukesh D. QSAR studies on chalcones and flavonoids as anti-tuberculosis agents using genetic function approximation (GFA) method. Chem Pharm Bull 2007;55:44–9. 10.1248/cpb.55.44 17202700

[39] Jarvill-Taylor KJ, Anderson RA, Graves DJ. A hydroxychalcone derived from cinnamon functions as a mimetic for insulin in 3T3-L1 adipocytes. J Am Coll Nutr 2001;20:327–36. 11506060 10.1080/07315724.2001.10719053

[40] Ovais S, Yaseen S, Bashir R, et al. Synthesis and anti-inflammatory activity of celecoxib like compounds. J Enzyme Inhib Med Chem 2013;28:1105–12. 22957719 10.3109/14756366.2012.710847

[41] Fylaktakidou KC, Hadjipavlou-Litina DJ, Litinas KE, et al. Recent developments in the chemistry and in the biological applications of amidoximes. Curr Pharm Des 2008;14:1001–47. 18473852 10.2174/138161208784139675

[42] Orlek BS, Blaney FE, Brown F, et al. Comparison of azabicyclic esters and oxadiazoles as ligands for the muscarinic receptor. J Med Chem 1991;34:2726–35. 1895293 10.1021/jm00113a009

[43] Bora RO, Dar B, Pradhan V, Farooqui M. [1, 2, 4]-Oxadiazoles: synthesis and biological applications. Mini-Rev Med Chem 2014;14:355–69. 24678879 10.2174/1389557514666140329200745

[44] Malamas MS, Sredy J, McCaleb M, et al. Antihyperglycemic activity of new 1,2,4-oxadiazolidine-3,5-diones. Eur J Med Chem 2001;36:31–42. 11231047 10.1016/s0223-5234(00)01191-0

[45] Ningaiah S, Bhadraiah UK, Keshavamurthy S, Javarasetty C. Novel pyrazoline amidoxime and their 1,2,4-oxadiazole analogues: synthesis and pharmacological screening. Bioorg Med Chem Lett 2013;23:4532–9. 23850201 10.1016/j.bmcl.2013.06.042

[46] Hankittichai P, Buacheen P, Pitchakarn P, et al. Artocarpus lakoocha extract inhibits LPS-induced inflammatory response in RAW 264.7 macrophage cells. Int J Mol Sci 2020;21:1355. 10.3390/ijms21041355 PMC7072914 32079307

[47] Dzoyem JP, Donfack AR, Tane P, et al. Inhibition of nitric oxide production in LPS-stimulated RAW 264.7 macrophages and 15-LOX activity by anthraquinones from *Pentas schimperi*. Planta Med 2016;82:1246–51. 27093247 10.1055/s-0042-104417

[48] Joo T, Sowndhararajan K, Hong S, et al. Inhibition of nitric oxide production in LPS-stimulated RAW 264.7 cells by stem bark of Ulmus pumila L. Saudi J Biol Sci 2014;21:427–35. 25313277 10.1016/j.sjbs.2014.04.003 PMC4191610

[49] Sekhar S, Sampath-Kumara KK, Niranjana SR, Prakash HS. Attenuation of reactive oxygen/nitrogen species with suppression of inducible nitric oxide synthase expression in RAW 264.7 macrophages by bark extract of *Buchanania lanzan*. Pharmacogn Mag 2015;11:283–91. 25829766 10.4103/0973-1296.153080 PMC4378125

[50] Kota DJ, Prabhakara KS, Toledano-Furman N, et al. Prostaglandin E2 indicates therapeutic efficacy of mesenchymal stem cells in experimental traumatic brain injury. Stem Cells 2017;35:1416–30. 28233425 10.1002/stem.2603

[51] Nossier ES, Fahmy HH, Khalifa NM, et al. Design and synthesis of novel pyrazole-substituted different nitrogenous heterocyclic ring systems as potential anti-inflammatory agents. Molecules 2017;22:512. 10.3390/molecules22040512 PMC6154115 28338602

[52] Pitchford LM, Smith JD, Abumrad NN, et al. Acute and 28-day repeated dose toxicity evaluations of 2-hydroxybenzylamine acetate in mice and rats. Regul Toxicol Pharmacol 2018;98:190–8. 30075181 10.1016/j.yrtph.2018.07.026

[53] Hallinan EA, Kramer SW, Houdek SC, et al. 4-Fluorinated L-lysine analogs as selective i-NOS inhibitors: methodology for introducing fluorine into the lysine side chain. Org Biomol Chem 2003;1:3527–34. 14599013 10.1039/b307563j

[54] Mohamed MFA, Youssif BGM, Shaykoon MSA, et al. Utilization of tetrahydrobenzo[4,5]thieno[2,3-d]pyrimidinone as a cap moiety in design of novel histone deacetylase inhibitors. Bioorg Chem 2019;91:103127. 31374527 10.1016/j.bioorg.2019.103127

[55] Abd El-kader AM, Mahmoud BK, Hajjar D, et al. Antiproliferative activity of new pentacyclic triterpene and a saponin from Gladiolus segetum Ker-Gawl corms supported by molecular docking study. RSC Adv 2020;10:22730–41. 10.1039/d0ra02775h PMC9054649 35514559

[56] Ibrahim TS, Sheha TA, Abo-Dya NE, et al. Design, synthesis and anticancer activity of novel valproic acid conjugates with improved histone deacetylase (HDAC) inhibitory activity. Bioorg Chem 2020;99:103797. 32247939 10.1016/j.bioorg.2020.103797

[57] Albayati MR, Mohamed MF, Moustafa AH. Optimization of the synthesis of het/aryl-amidoximes using an efficient green chemistry. Synth Comm 2020;50:1–15.

[58] Ali H, Khan A, Ali J, et al. Attenuation of LPS-induced acute lung injury by continentalic acid in rodents through inhibition of inflammatory mediators correlates with increased Nrf2 protein expression. BMC Pharmacol Toxicol 2020;21:81. 33239093 10.1186/s40360-020-00458-7 PMC7687815

[59] Chan PM, Tan YS, Chua KH, et al. Attenuation of inflammatory mediators (TNF-alpha and nitric oxide) and up-regulation of IL-10 by wild and domesticated basidiocarps of *Amauroderma rugosum* (Blume & T. Nees) torrend in LPS-stimulated RAW264.7 cells. PLoS One 2015;10:e0139593. 26427053 10.1371/journal.pone.0139593 PMC4591274

[60] Soufli I, Toumi R, Rafa H, Touil-Boukoffa C. Overview of cytokines and nitric oxide involvement in immuno-pathogenesis of inflammatory bowel diseases. World J Gastrointest Pharmacol Ther 2016;7:353–60. 27602236 10.4292/wjgpt.v7.i3.353 PMC4986402

[61] Du Q, Luo J, Yang M-Q, et al. iNOS/NO is required for IRF1 activation in response to liver ischemia-reperfusion in mice. Mol Med 2020;26:56. 32517688 10.1186/s10020-020-00182-2 PMC7285570

[62] Lamie PF, Philoppes JN. 2-Thiopyrimidine/chalcone hybrids: design, synthesis, ADMET prediction, and anticancer evaluation as STAT3/STAT5a inhibitors. J Enzyme Inhib Med Chem 2020;35:864–79. 32208772 10.1080/14756366.2020.1740922 PMC7144330

[63] Lipinski CA, Lombardo F, Dominy BW, Feeney PJ. Experimental and computational approaches to estimate solubility and permeability in drug discovery and development settings. Adv Drug Deliv Rev 2001;46:3–26. 11259830 10.1016/s0169-409x(00)00129-0

[64] Veber DF, Johnson SR, Cheng HY, et al. Molecular properties that influence the oral bioavailability of drug candidates. J Med Chem 2002;45:2615–23. 12036371 10.1021/jm020017n

[65] Wadapurkar RM, Shilpa M, Katti AKS, Sulochana M. In silico drug design for Staphylococcus aureus and development of host-pathogen interaction network. Inf Med Unlocked 2018;10:58–70.

[66] Wang R, Fu Y, Lai L. A new atom-additive method for calculating partition coefficients. J Chem Inf Comput Sci 1997;37:615–21.

