# Supplementary material for: Novel chalcone/aryl carboximidamide hybrids as potent anti-inflammatory via inhibition of prostaglandin E2 and inducible NO synthase activities: design, synthesis, molecular docking studies and ADMET prediction
Source: J Enzyme Inhib Med Chem. 2021 May 24;36(1):1067–78. doi: 10.1080/14756366.2021.1929201 (PMC8158245; doi:10.1080/14756366.2021.1929201)

Supplementary file

Novel chalcone/aryl carboximidamide hybrids as potent anti-inflammatory via inhibition of prostaglandin E2 and inducible NO synthase activities: Design, synthesis, molecular docking studies and ADMET prediction

Tarek S. Ibrahim [1,2,*], Amr H. Moustafa [3], Ahmad J. Almalki [1], Rasha M. Allam [4], Abdulhamid Althagafi [5], Shadab Md [6], Mamdouh F. A. Mohamed [7,*]

[1] Department of Pharmaceutical Chemistry, Faculty of Pharmacy, King Abdulaziz University, Jeddah, 21589, Saudi Arabia.
[2] Department of Pharmaceutical Organic Chemistry, Faculty of Pharmacy, Zagazig University, Zagazig, 44519, Egypt.
[3] Department of Chemistry, Faculty of Science, Sohag University, Sohag 82524, Egypt.
[4] Pharmacology Department, National Research Centre, Cairo, 12622, Egypt.
[5] Department of Pharmacy Practice, Faculty of Pharmacy, King Abdulaziz University, Jeddah, 21589, Saudi Arabia.
[6] Department of Pharmaceutics, Faculty of pharmacy, King Abdulaziz University, Jeddah, 21589, Saudi Arabia.
[7] Department of Pharmaceutical Chemistry, Faculty of Pharmacy, Sohag University, 82524 Sohag, Egypt.

* Correspondence: Tarek S. Ibrahim, Associate Professor, Department of Pharmaceutical Chemistry, Faculty of Pharmacy, King Abdulaziz University, Jeddah, 21589, Saudi Arabia. **Tel.:** +966535846571. **E-mail**: tmabrahem@kau.edu.sa, tarekeldeeb1976@yahoo.com

* Correspondence: Mamdouh F. A. Mohamed, Ph.D, Department of Pharmaceutical Chemistry, Faculty of Pharmacy, Sohag University, 82524-Sohag, Egypt. **Tel.:** (002)-01018384461 **E-mail**: mamdouh.fawzi@pharm.sohag.edu.eg, mamdouhfawzy3@yahoo.com

Docking methodology **S2.**

^{1}H NMR and ^{13}C NMR **S3-S13**

5.3. Docking methodology

Discovery Studio 2.5 software (Accelrys Inc., San Diego, CA, USA) was used for docking analysis. Fully automated docking tool using "Dock ligands (CDOCKER)" protocol running on Intel (R) core (TM) i32370 CPU @ 2.4 GHz 2.4 GHz, RAM Memory 2 GB under the Windows 7.0 system. The crystal structures of iNOS protein (PDB code: 1r35) was downloaded from protein data bank [1-5]. The docked compounds were built using Chem. 3D ultra 12.0 software [Chemical Structure Drawing Standard; Cambridge Soft corporation, USA (2010)], and copied to Discovery Studio 2.5 software. Automatic protein preparation module was used applying MMFF94 force field. The binding site sphere has been defined automatically by the software. Now the above prepared receptor is given as input for "input receptor molecule" parameter in the CDOCKER protocol parameter explorer. Force fields are applied on compounds **4a** and **4d** to get the minimum lowest energy structure. The obtained poses were studied and the poses showing best ligand–iNOS interactions were selected and used for CDOCKER energy (protein-ligand interaction energies) calculations. Receptor–ligand interactions of the complexes were examined in 2D and 3D styles bank.

[1] E.A. Hallinan, S.W. Kramer, S.C. Houdek, W.M. Moore, G.M. Jerome, D.P. Spangler, A.M. Stevens, H.S. Shieh, P.T. Manning, B.S. Pitzele, 4-Fluorinated L-lysine analogs as selective i-NOS inhibitors: methodology for introducing fluorine into the lysine side chain, Org Biomol Chem 1(20) (2003) 3527-34.
[2] L. Ma, H. Pei, L. Lei, L. He, J. Chen, X. Liang, A. Peng, H. Ye, M. Xiang, L. Chen, Structural exploration, synthesis and pharmacological evaluation of novel 5-benzylidenethiazolidine-2,4-dione derivatives as iNOS inhibitors against inflammatory diseases, Eur J Med Chem 92 (2015) 178-90.
[3] M.F.A. Mohamed, B.G.M. Youssif, M.S.A. Shaykoon, M.H. Abdelrahman, B.E.M. Elsadek, A.S. Aboraia, G.E.A. Abuo-Rahma, Utilization of tetrahydrobenzo[4,5]thieno[2,3-d]pyrimidinone as a cap moiety in design of novel histone deacetylase inhibitors, Bioorg Chem 91 (2019) 103127.
[4] A.M. Abd El-kader, B.K. Mahmoud, D. Hajjar, M.F. Mohamed, A.M. Hayallah, U.R. Abdelmohsen, Antiproliferative activity of new pentacyclic triterpene and a saponin from Gladiolus segetum Ker-Gawl corms supported by molecular docking study, RSC Advances 10(38) (2020) 22730-22741.
[5] T.S. Ibrahim, T.A. Sheha, N.E. Abo-Dya, M.A. AlAwadh, N.A. Alhakamy, Z.K. Abdel-Samii, S.S. Panda, G.E.A. Abuo-Rahma, M.F.A. Mohamed, Design, synthesis and anticancer activity of novel valproic acid conjugates with improved histone deacetylase (HDAC) inhibitory activity, Bioorg Chem 99 (2020) 103797.

1. ^{1}H and ^{13}C NMR Spectra of 4a:

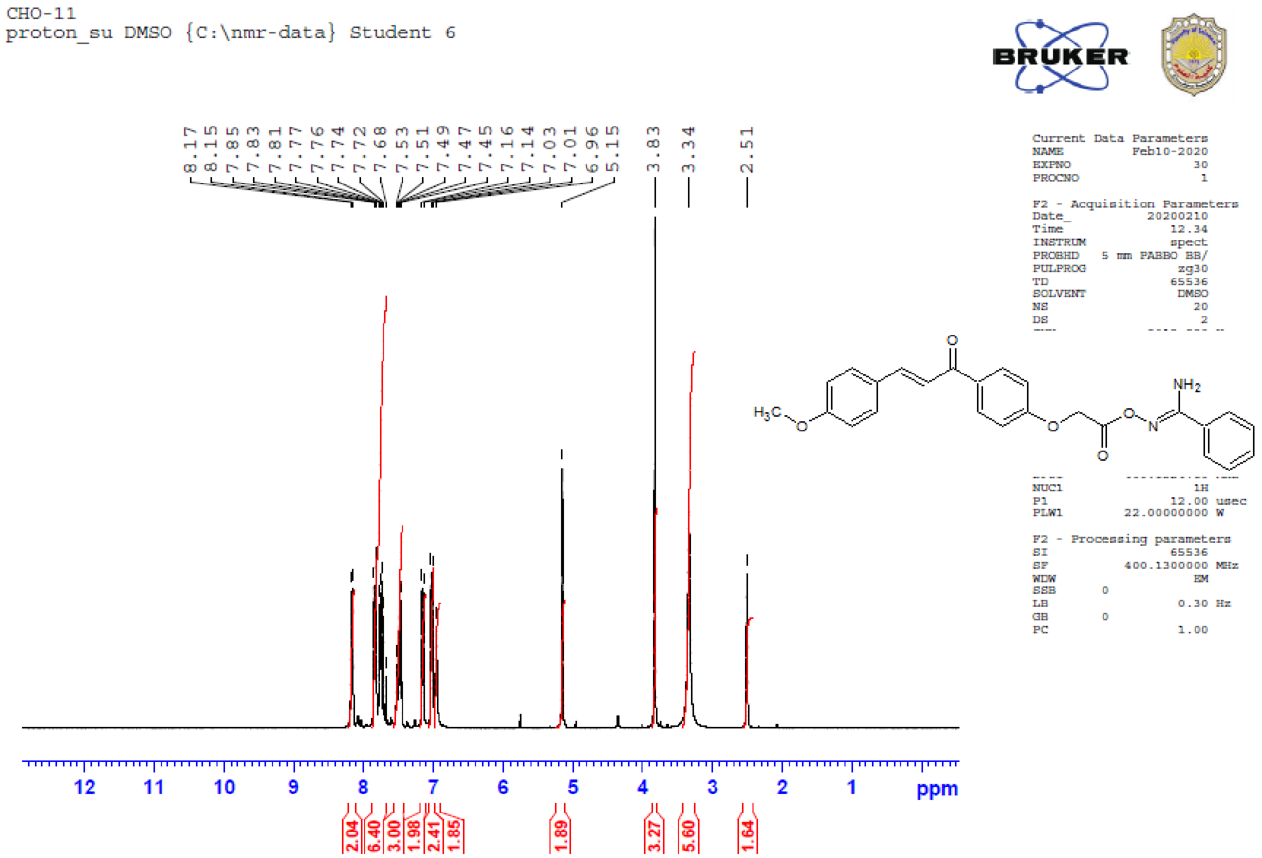

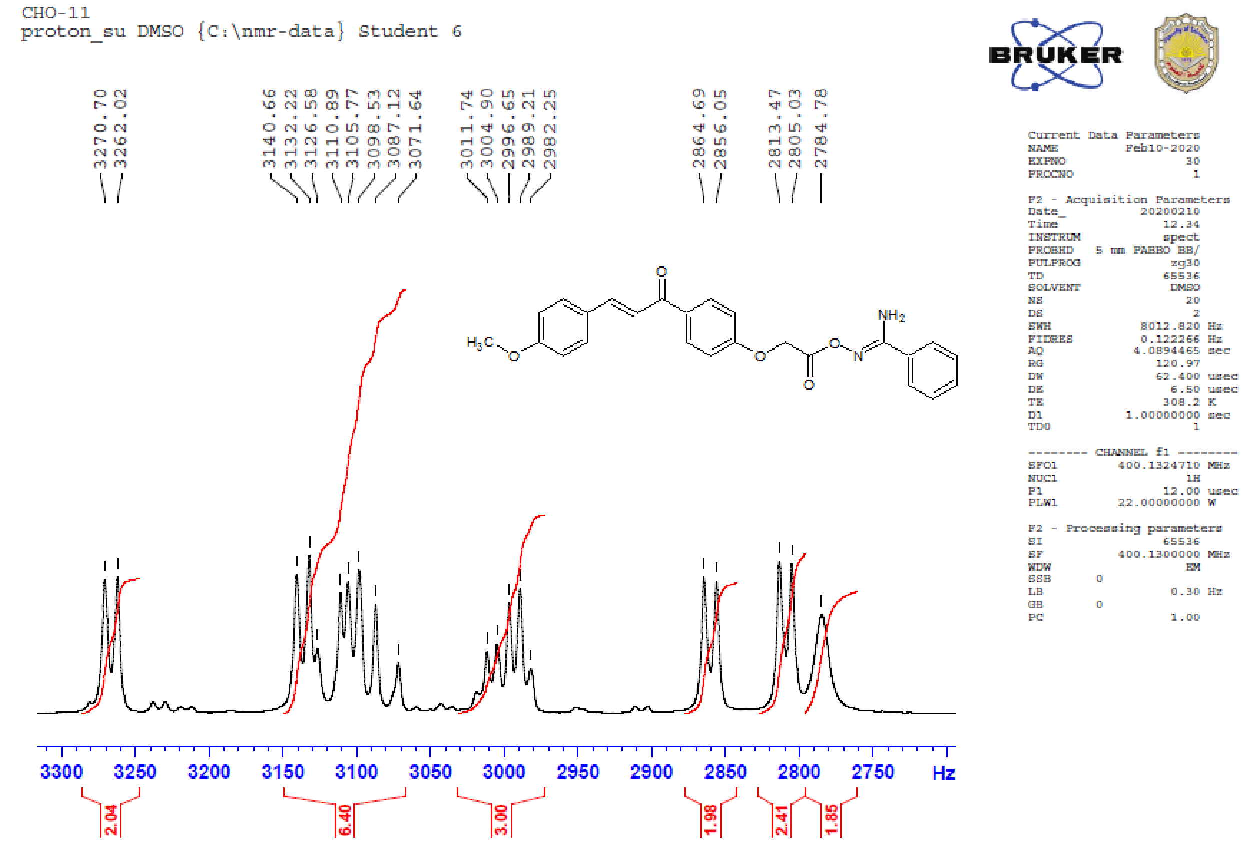

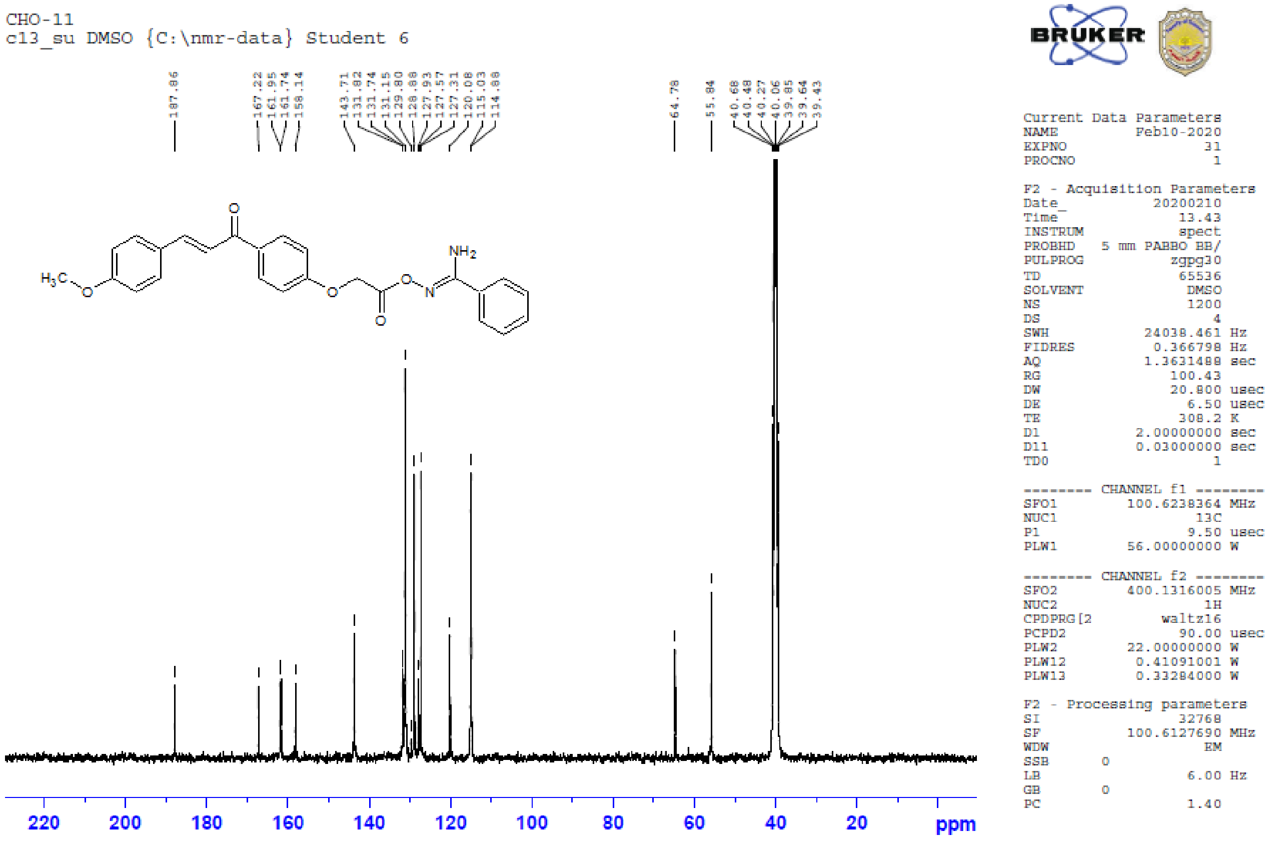

2. ^{1}H and ^{13}C NMR Spectra of 4b:

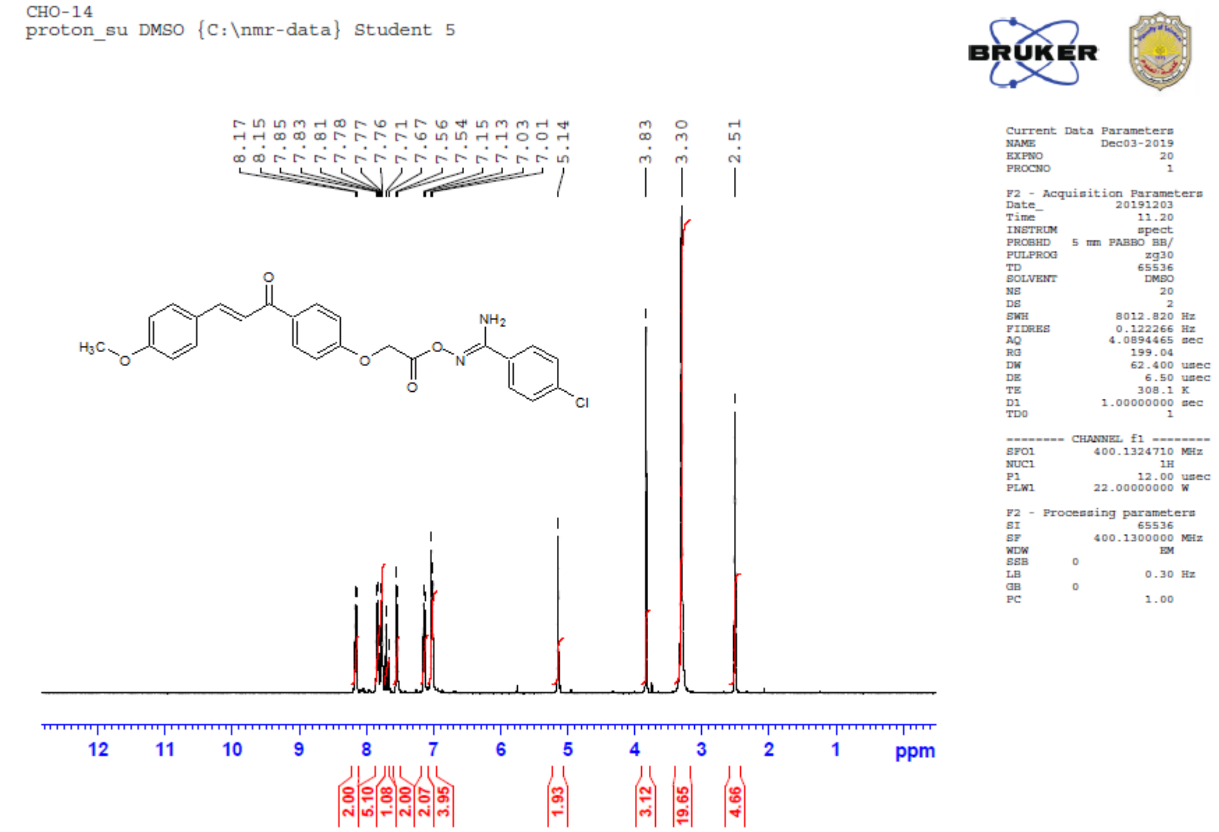
CHO-14
proton_su DMSO {C:\nmr-data} Student 5
8.17
8.15
7.85
7.83
7.81
7.78
7.77
7.76
7.71
7.67
7.56
7.54
7.15
7.13
7.03
7.01
5.14
3.83
3.30
2.51
BRUKER
Current Data Parameters
NAME Dec03-2019
EXPNO 20
PROCNO 1
F2 - Acquisition Parameters
Date_ 20191203
Time 11.20
INSTRUM spect
PROBHD 5 mm PABBO BB/
PULPROG zg30
TD 65536
SOLVENT DMSO
NS 20
DS 2
SWH 8012.820 Hz
FIDRES 0.122266 Hz
AQ 4.0894465 sec
RG 199.04
DW 62.400 usec
DE 6.50 usec
TE 308.1 K
D1 1.00000000 sec
TD0 1
======== CHANNEL f1 ========
SFO1 400.1324710 MHz
NUC1 1H
P1 12.00 usec
PLW1 22.00000000 W
F2 - Processing parameters
SI 65536
SF 400.1300000 MHz
WDW EM
SSB 0
LB 0.30 Hz
GB 0
PC 1.00
O
NH2
H3C
O
O
O
N
O
Cl
12
11
10
9
8
7
6
5
4
3
2
1
ppm
2.00
5.10
1.08
2.00
2.07
3.95
1.93
3.12
19.65
4.66

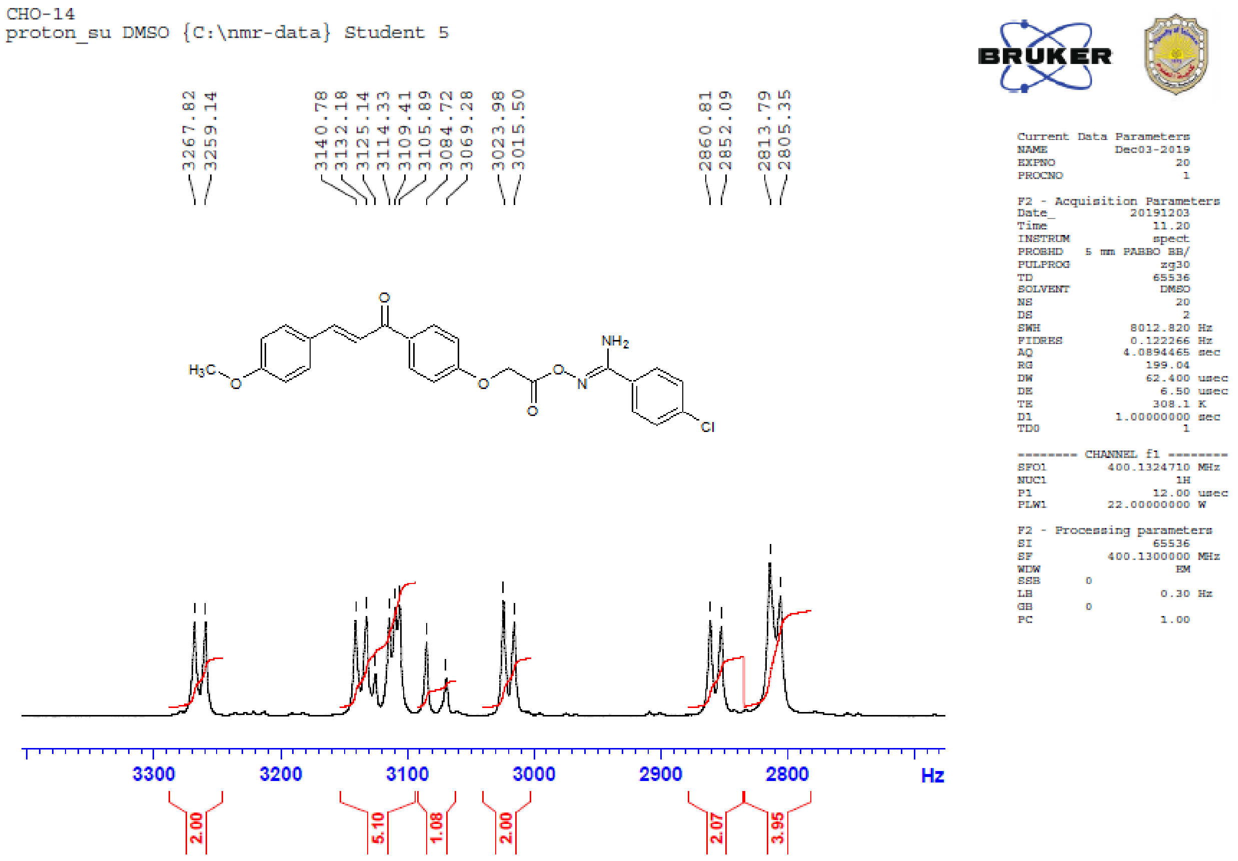
CHO-14
proton_su DMSO {C:\nmr-data} Student 5
3267.82
3259.14
3140.78
3132.18
3125.14
3114.33
3109.41
3105.89
3084.72
3069.28
3023.98
3015.50
2860.81
2852.09
2813.79
2805.35
BRUKER
Current Data Parameters
NAME Dec03-2019
EXPNO 20
PROCNO 1
F2 - Acquisition Parameters
Date_ 20191203
Time 11.20
INSTRUM spect
PROBHD 5 mm PABBO BB/
PULPROG zg30
TD 65536
SOLVENT DMSO
NS 20
DS 2
SWH 8012.820 Hz
FIDRES 0.122266 Hz
AQ 4.0894465 sec
RG 199.04
DW 62.400 usec
DE 6.50 usec
TE 308.1 K
D1 1.00000000 sec
TD0 1
======== CHANNEL f1 ========
SFO1 400.1324710 MHz
NUC1 1H
P1 12.00 usec
PLW1 22.00000000 W
F2 - Processing parameters
SI 65536
SF 400.1300000 MHz
WDW EM
SSB 0
LB 0.30 Hz
GB 0
PC 1.00
O
NH2
H3C
O
O
O
N
O
Cl
3300
3200
3100
3000
2900
2800
Hz
2.00
5.10
1.08
2.00
2.07
3.95

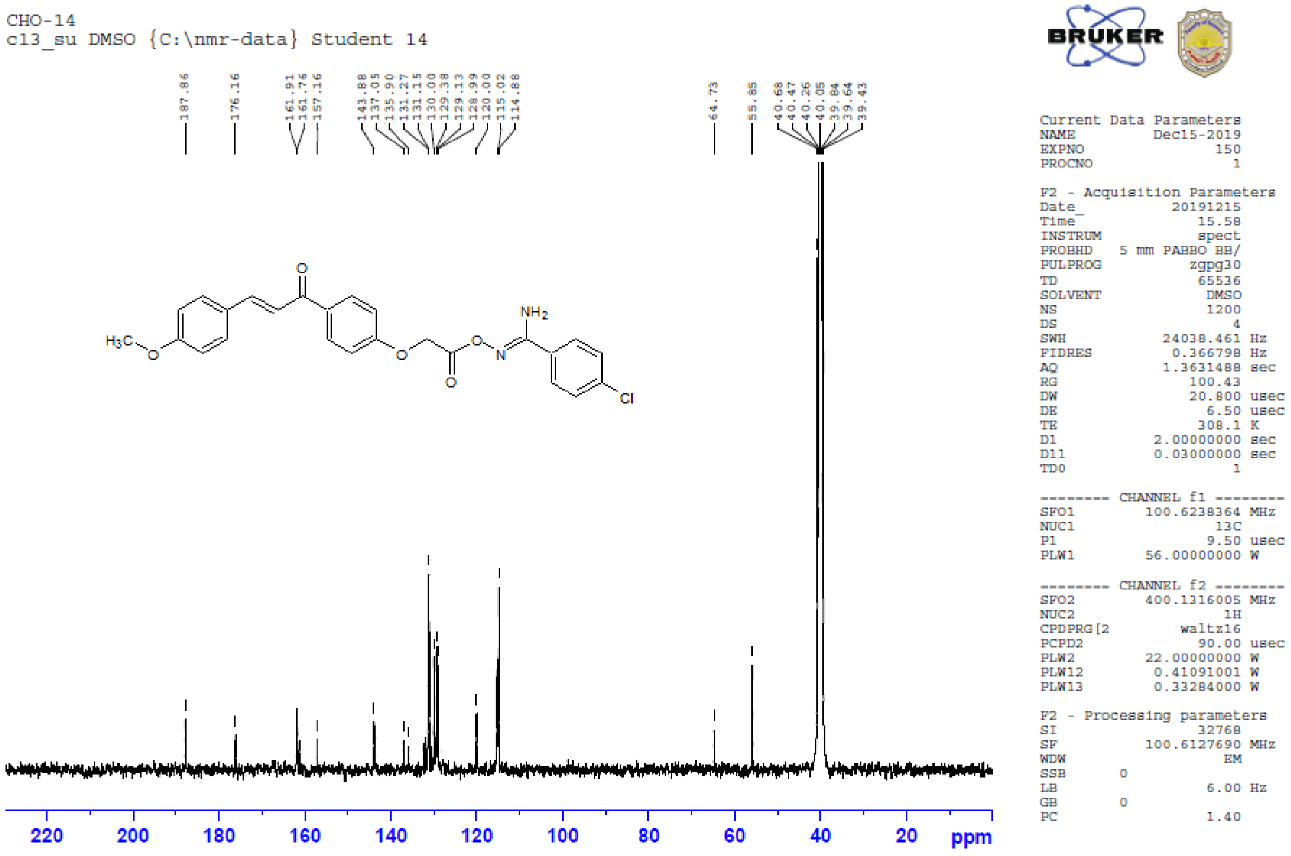

3. ^{1}H, ^{13}C NMR and Dept-135 Spectra of 4c:

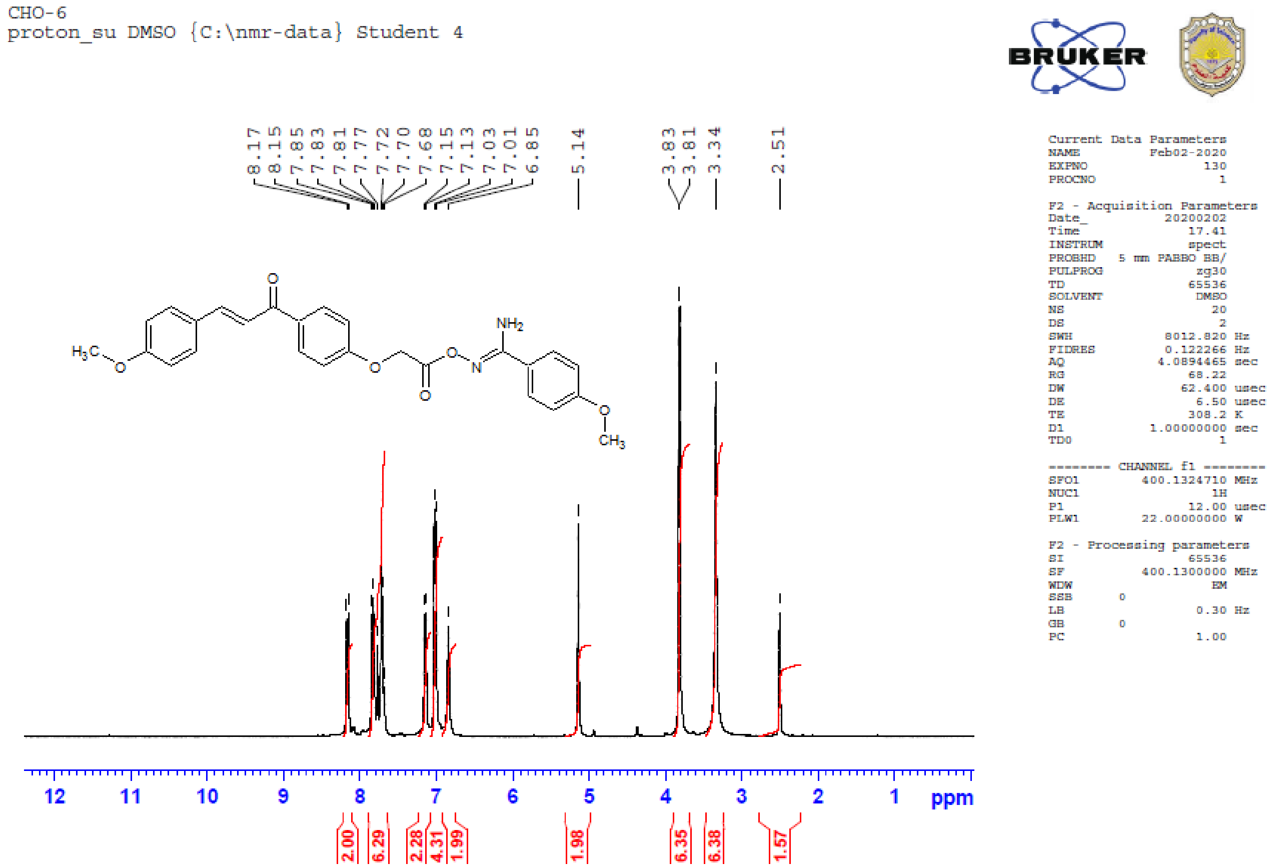

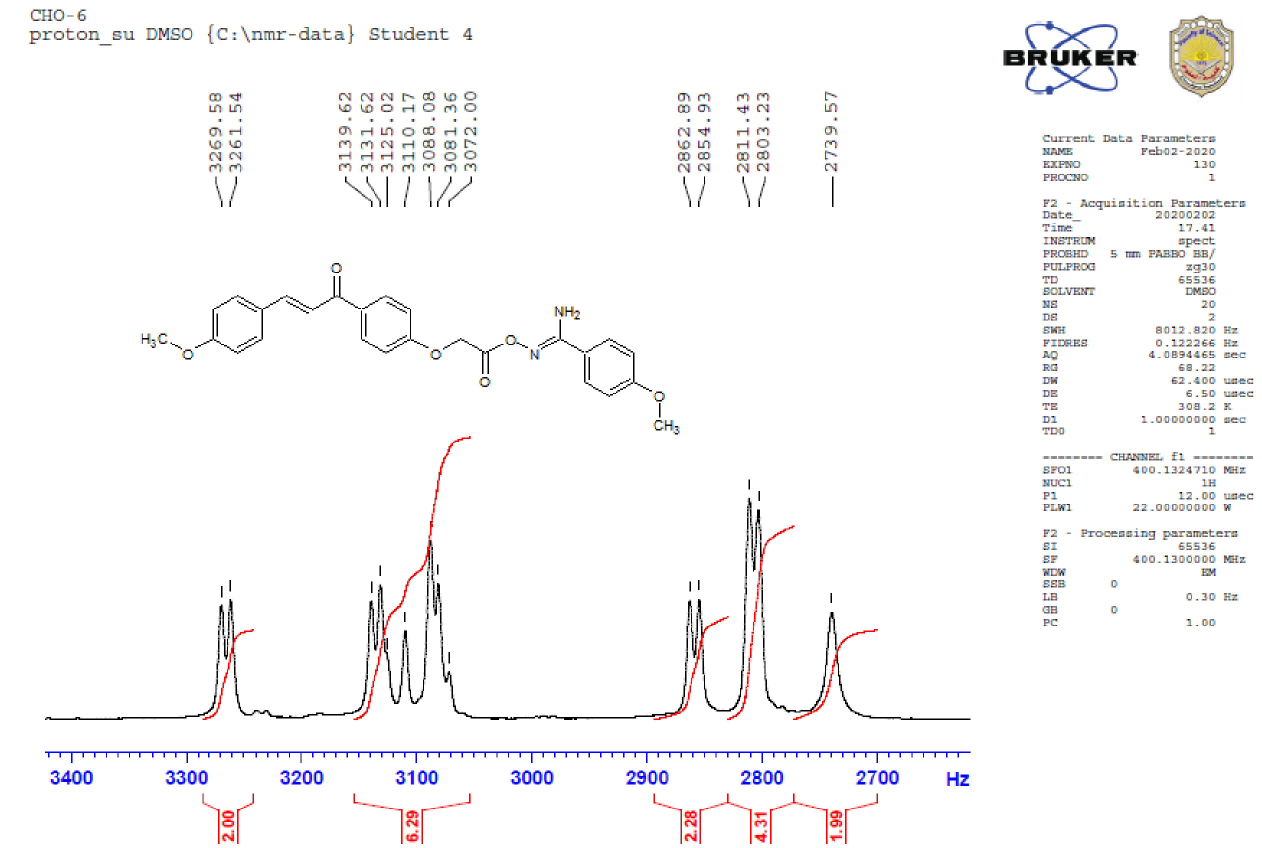
CHO-6
proton_su DMSO {C:\nmr-data} Student 4
3269.58
3261.54
3139.62
3131.62
3125.02
3110.17
3088.08
3081.36
3072.00
2862.89
2854.93
2811.43
2803.23
2739.57
NH2
H3C
O
N
CH3
Hz
2.00
6.29
2.28
4.31
1.99

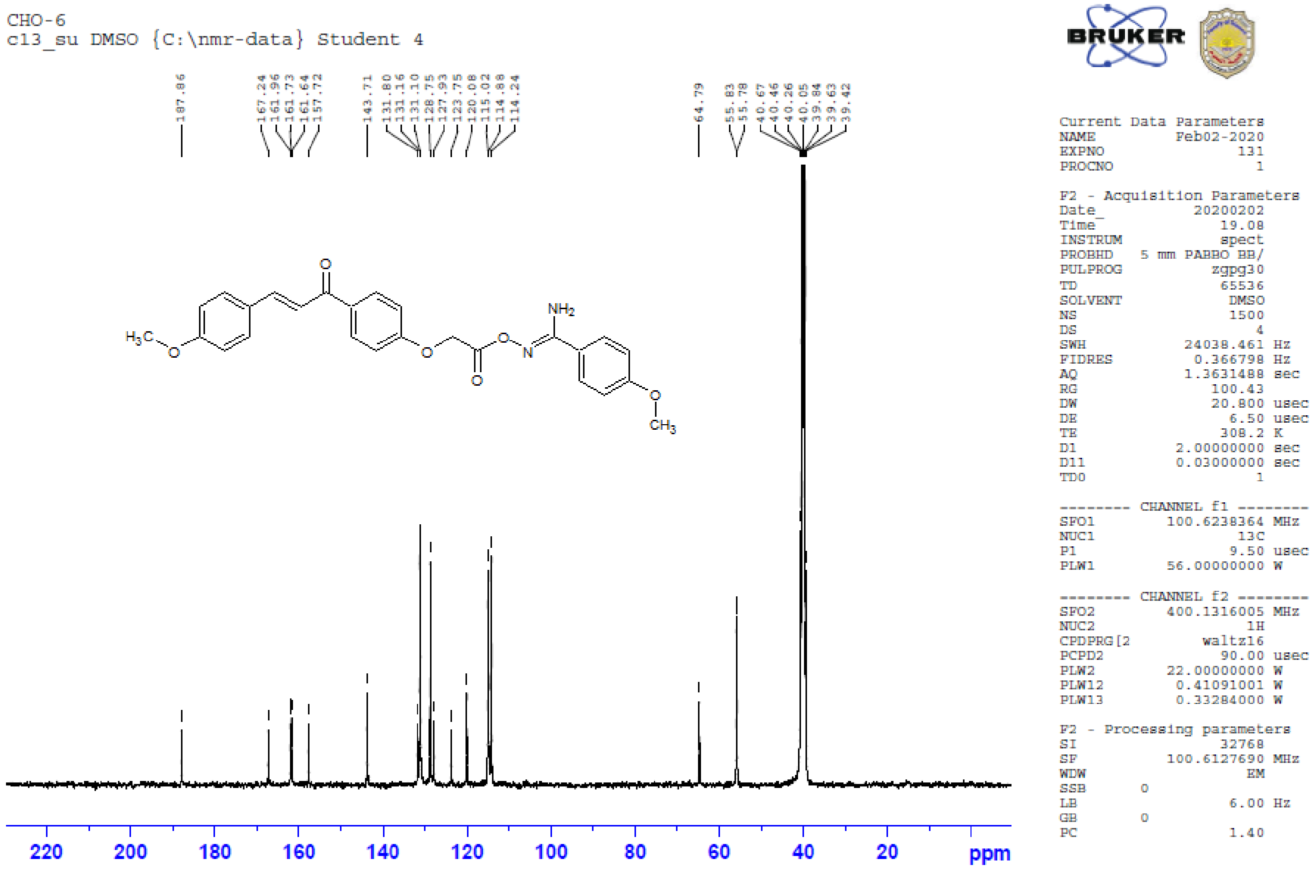
CHO-6
c13_su DMSO {C:\nmr-data} Student 4
187.86
167.24
161.96
161.73
161.64
157.72
143.71
131.80
131.16
131.10
128.75
127.93
123.75
120.08
115.02
114.88
114.24
64.79
55.83
55.78
40.67
40.46
40.26
40.05
39.84
39.63
39.42
NH2
H3C
O
N
CH3
ppm

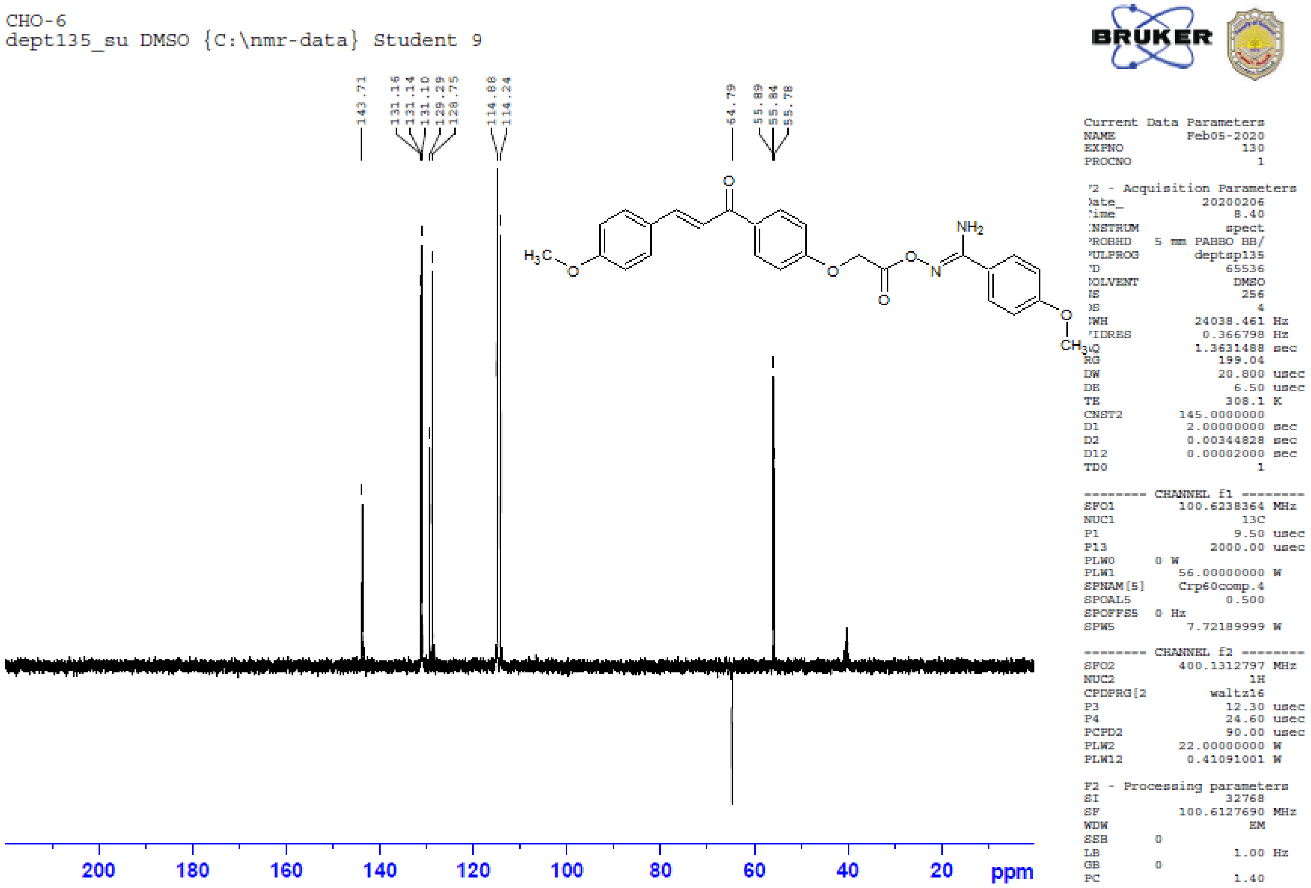

4. ^{1}H and ^{13}C NMR Spectra of 4d:

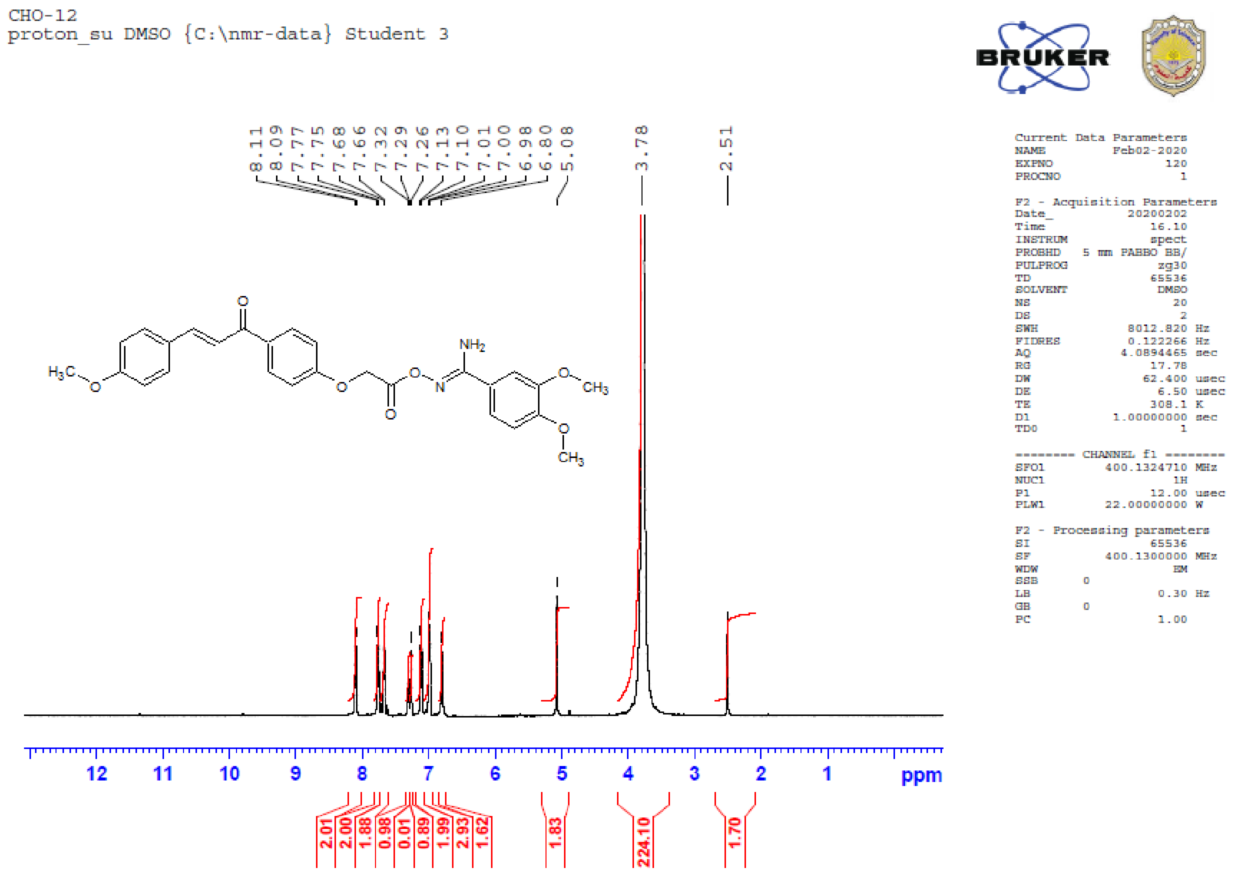
CHO-12
proton_su DMSO {C:\nmr-data} Student 3
BRUKER
8.11 8.09 7.77 7.75 7.68 7.66 7.32 7.29 7.26 7.13 7.10 7.01 7.00 6.98 6.80 5.08 3.78 2.51
Current Data Parameters
NAME Feb02-2020
EXPNO 120
PROCNO 1
F2 - Acquisition Parameters
Date_ 20200202
Time 16.10
INSTRUM spect
PROBHD 5 mm PABBO BB/
PULPROG zg30
TD 65536
SOLVENT DMSO
NS 20
DS 2
SWH 8012.820 Hz
FIDRES 0.122266 Hz
AQ 4.0894465 sec
RG 17.78
DW 62.400 usec
DE 6.50 usec
TE 308.1 K
D1 1.00000000 sec
TD0 1
======== CHANNEL f1 ========
SFO1 400.1324710 MHz
NUC1 1H
P1 12.00 usec
PLW1 22.00000000 W
F2 - Processing parameters
SI 65536
SF 400.1300000 MHz
WDW EM
SSB 0
LB 0.30 Hz
GB 0
PC 1.00
12 11 10 9 8 7 6 5 4 3 2 1 ppm
2.01 2.00 1.88 0.98 0.01 0.89 1.99 2.93 1.62 1.83 224.10 1.70

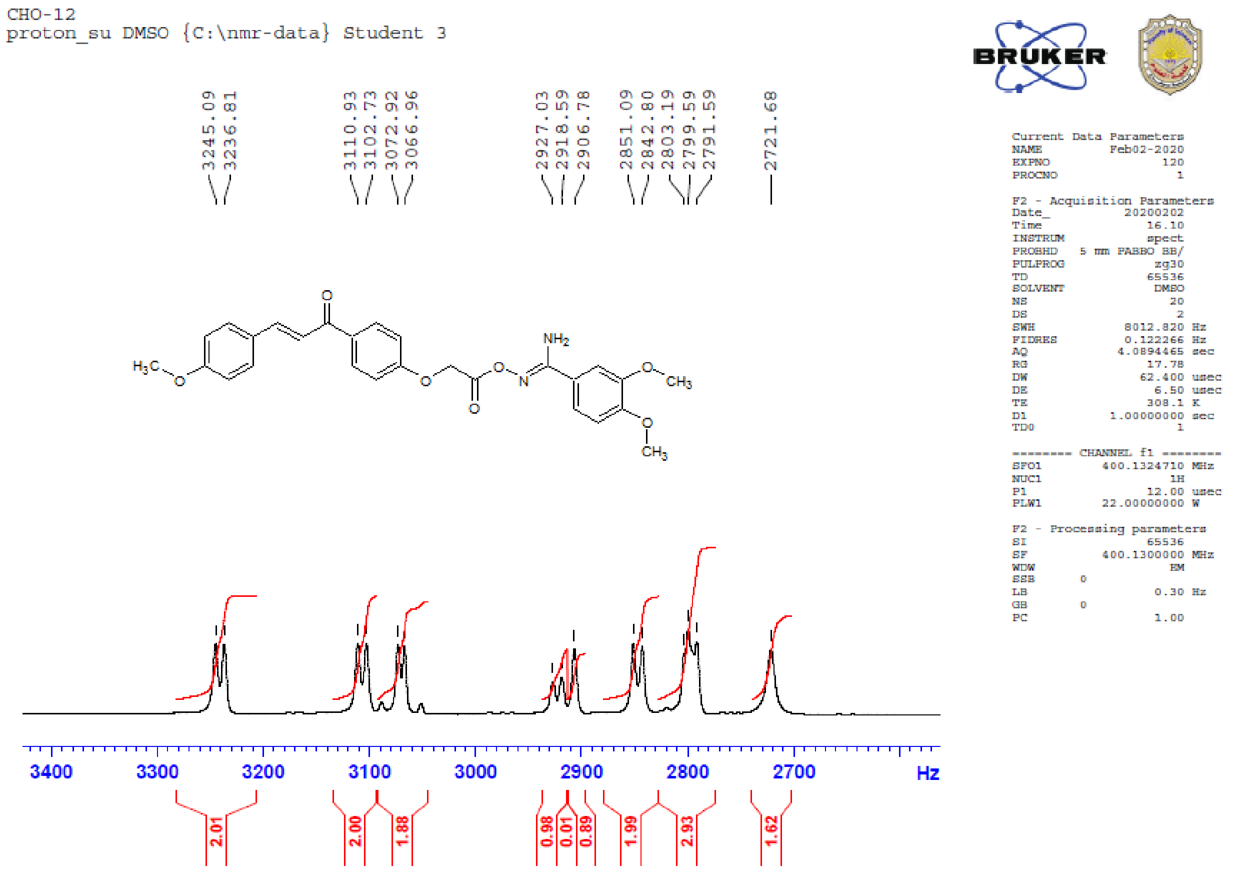
CHO-12
proton_su DMSO {C:\nmr-data} Student 3
BRUKER
3245.09 3236.81 3110.93 3102.73 3072.92 3066.96 2927.03 2918.59 2906.78 2851.09 2842.80 2803.19 2799.59 2791.59 2721.68
Current Data Parameters
NAME Feb02-2020
EXPNO 120
PROCNO 1
F2 - Acquisition Parameters
Date_ 20200202
Time 16.10
INSTRUM spect
PROBHD 5 mm PABBO BB/
PULPROG zg30
TD 65536
SOLVENT DMSO
NS 20
DS 2
SWH 8012.820 Hz
FIDRES 0.122266 Hz
AQ 4.0894465 sec
RG 17.78
DW 62.400 usec
DE 6.50 usec
TE 308.1 K
D1 1.00000000 sec
TD0 1
======== CHANNEL f1 ========
SFO1 400.1324710 MHz
NUC1 1H
P1 12.00 usec
PLW1 22.00000000 W
F2 - Processing parameters
SI 65536
SF 400.1300000 MHz
WDW EM
SSB 0
LB 0.30 Hz
GB 0
PC 1.00
3400 3300 3200 3100 3000 2900 2800 2700 Hz
2.01 2.00 1.88 0.98 0.01 0.89 1.99 2.93 1.62

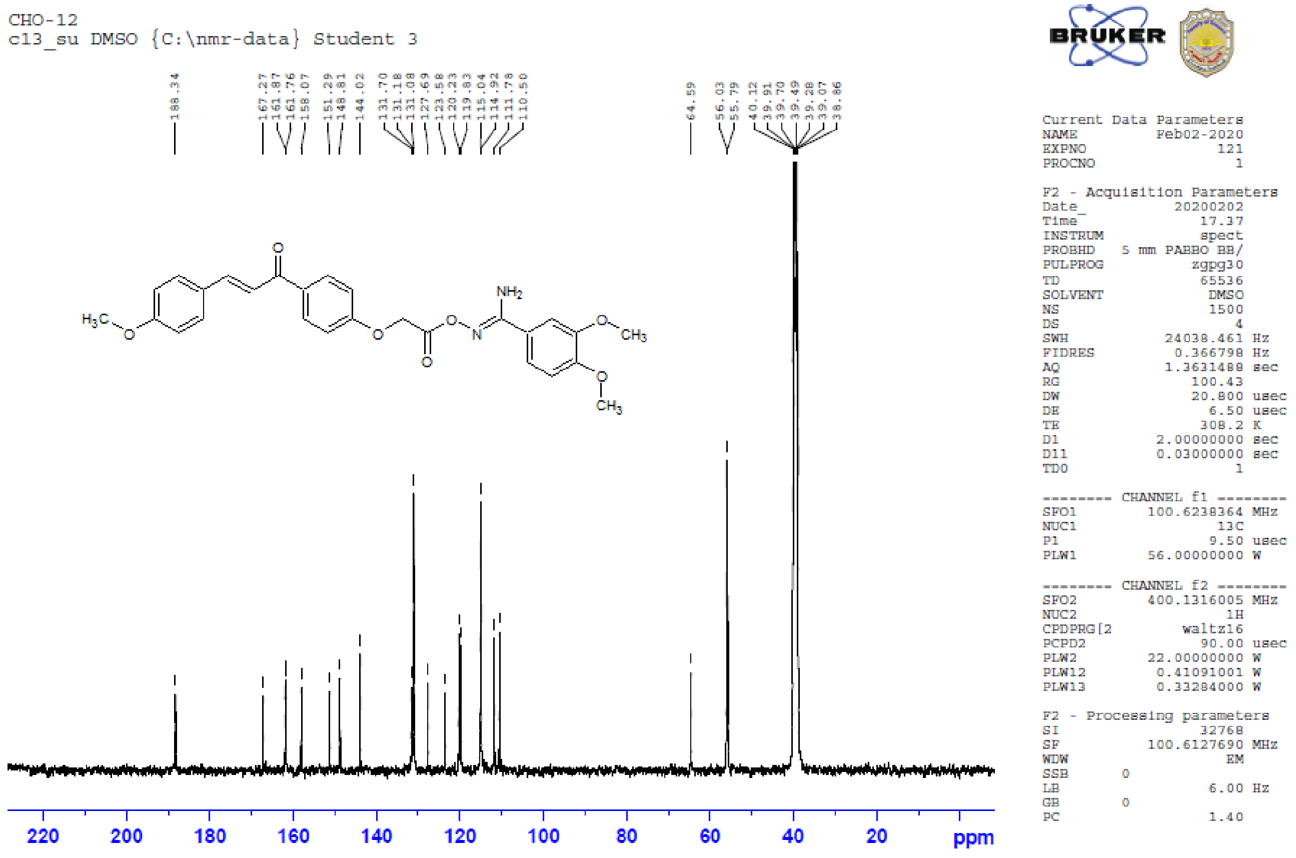

5. ^{1}H and ^{13}C NMR Spectra of 4e:

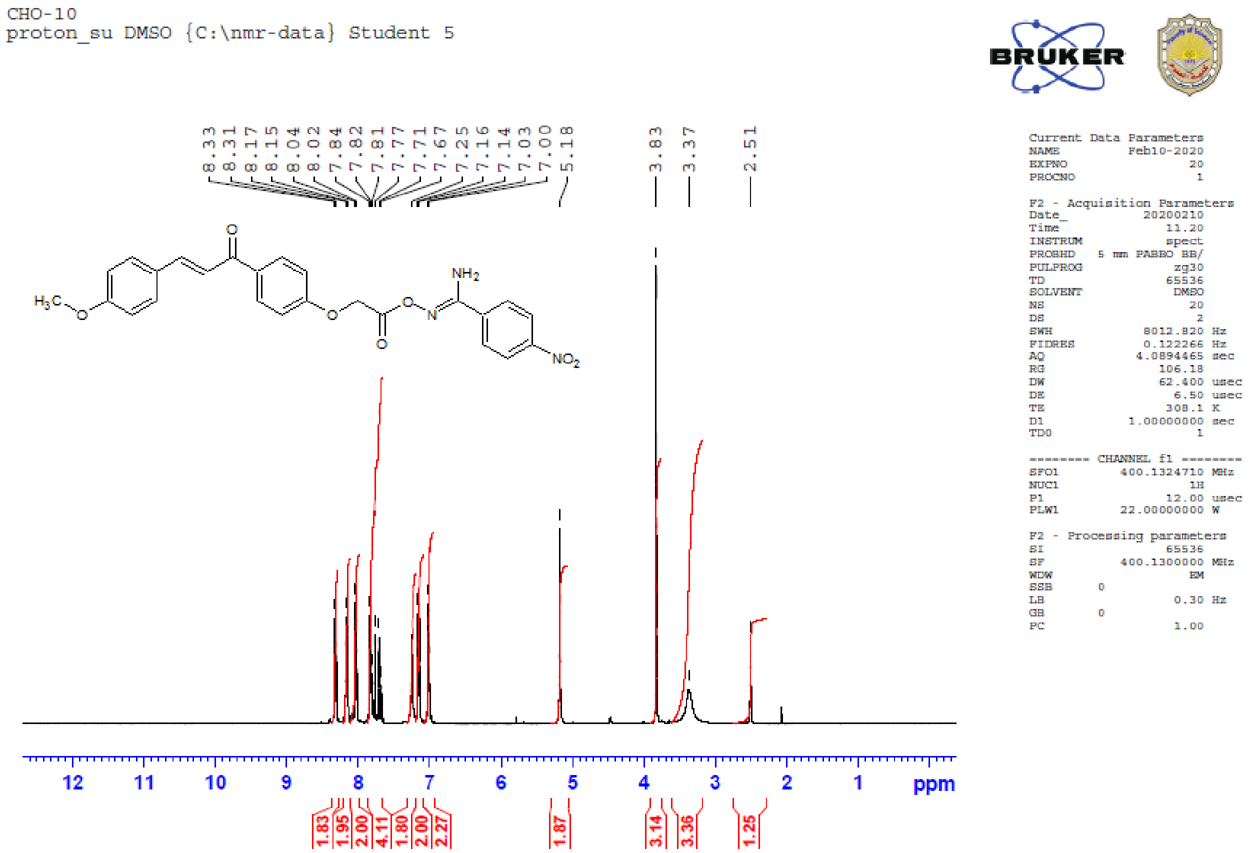

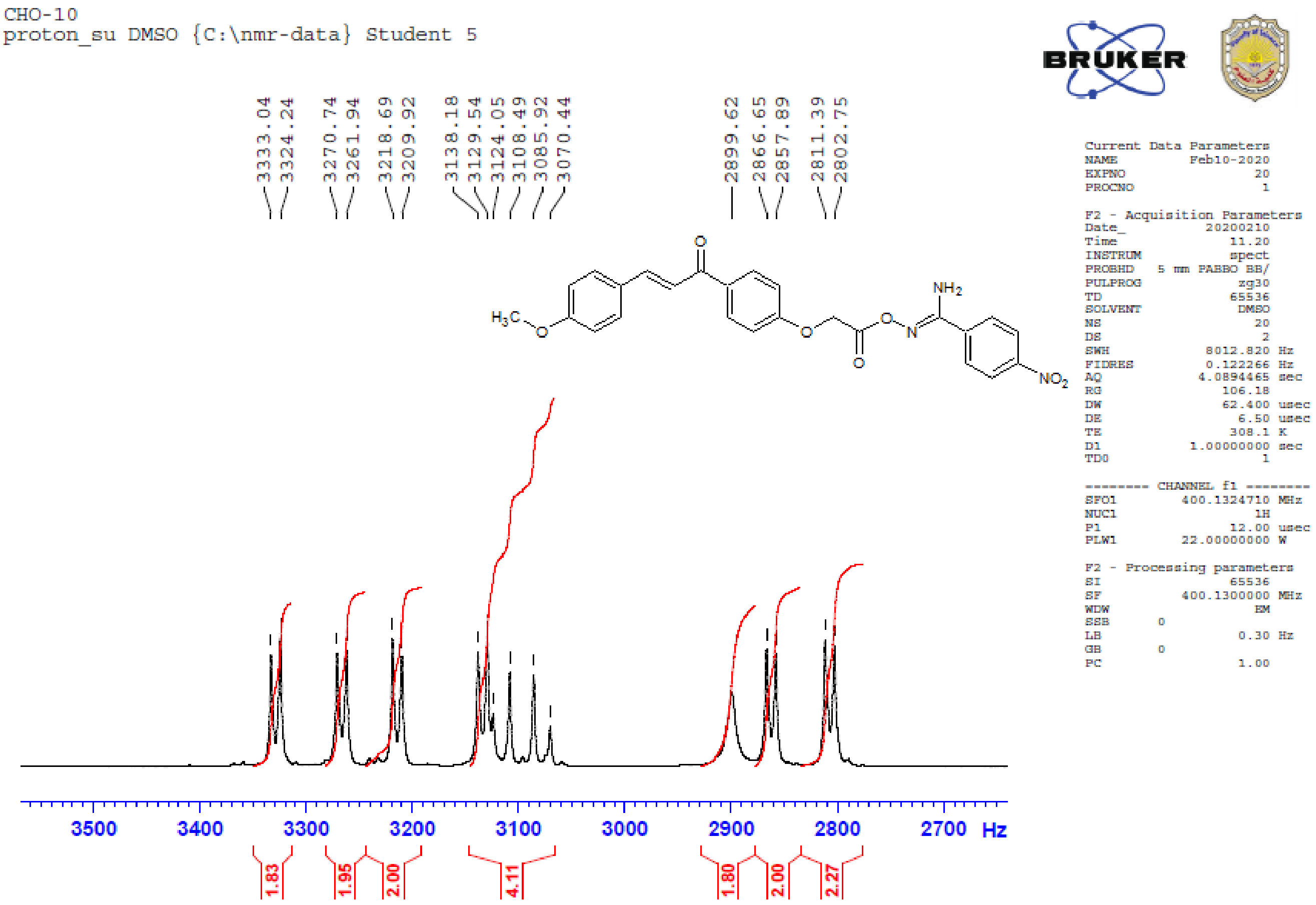

CHO-10
proton_su DMSO {C:\nmr-data} Student 5
BRUKER
Current Data Parameters
NAME Feb10-2020
EXPNO 20
PROCNO 1
F2 - Acquisition Parameters
Date_ 20200210
Time 11.20
INSTRUM spect
PROBHD 5 mm PABBO BB/
PULPROG zg30
TD 65536
SOLVENT DMSO
NS 20
DS 2
SWH 8012.820 Hz
FIDRES 0.122266 Hz
AQ 4.0894465 sec
RG 106.18
DW 62.400 usec
DE 6.50 usec
TE 308.1 K
D1 1.00000000 sec
TD0 1
======== CHANNEL f1 ========
SFO1 400.1324710 MHz
NUC1 1H
P1 12.00 usec
PLW1 22.00000000 W
F2 - Processing parameters
SI 65536
SF 400.1300000 MHz
WDW EM
SSB 0
LB 0.30 Hz
GB 0
PC 1.00
3333.04
3324.24
3270.74
3261.94
3218.69
3209.92
3138.18
3129.54
3124.05
3108.49
3085.92
3070.44
2899.62
2866.65
2857.89
2811.39
2802.75
H3C
O
NH2
NO2
3500
3400
3300
3200
3100
3000
2900
2800
2700
Hz
1.83
1.95
2.00
4.11
1.80
2.00
2.27

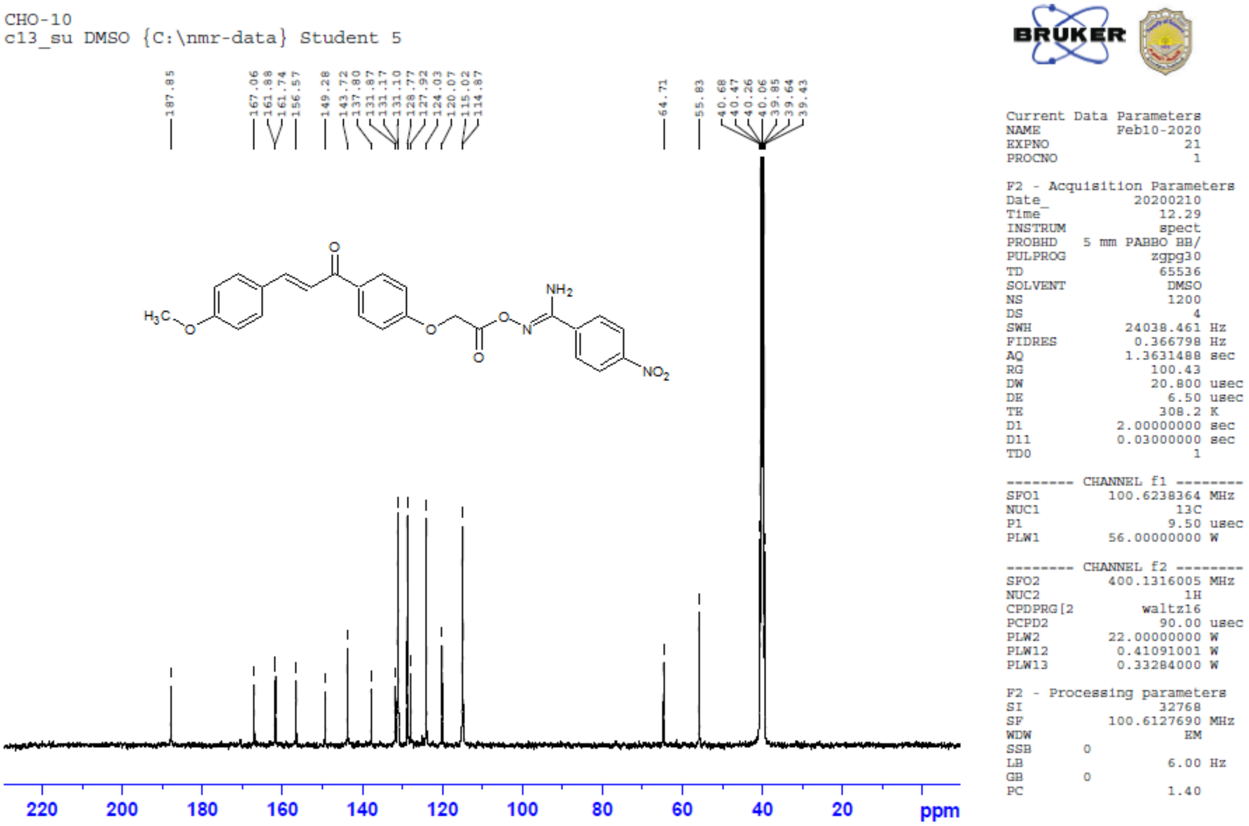

CHO-10
c13_su DMSO {C:\nmr-data} Student 5
BRUKER
Current Data Parameters
NAME Feb10-2020
EXPNO 21
PROCNO 1
F2 - Acquisition Parameters
Date_ 20200210
Time 12.29
INSTRUM spect
PROBHD 5 mm PABBO BB/
PULPROG zgpg30
TD 65536
SOLVENT DMSO
NS 1200
DS 4
SWH 24038.461 Hz
FIDRES 0.366798 Hz
AQ 1.3631488 sec
RG 100.43
DW 20.800 usec
DE 6.50 usec
TE 308.2 K
D1 2.00000000 sec
D11 0.03000000 sec
TD0 1
======== CHANNEL f1 ========
SFO1 100.6238364 MHz
NUC1 13C
P1 9.50 usec
PLW1 56.00000000 W
======== CHANNEL f2 ========
SFO2 400.1316005 MHz
NUC2 1H
CPDPRG[2 waltz16
PCPD2 90.00 usec
PLW2 22.00000000 W
PLW12 0.41091001 W
PLW13 0.33284000 W
F2 - Processing parameters
SI 32768
SF 100.6127690 MHz
WDW EM
SSB 0
LB 6.00 Hz
GB 0
PC 1.40
187.85
167.06
161.88
161.74
156.57
149.28
143.72
137.80
131.87
131.17
131.10
128.77
127.92
124.03
120.07
115.02
114.87
64.71
55.83
40.68
40.47
40.26
40.06
39.85
39.64
39.43
H3C
O
NH2
NO2
220
200
180
160
140
120
100
80
60
40
20
ppm

6. ^{1}H and ^{13}C NMR Spectra of 4f:

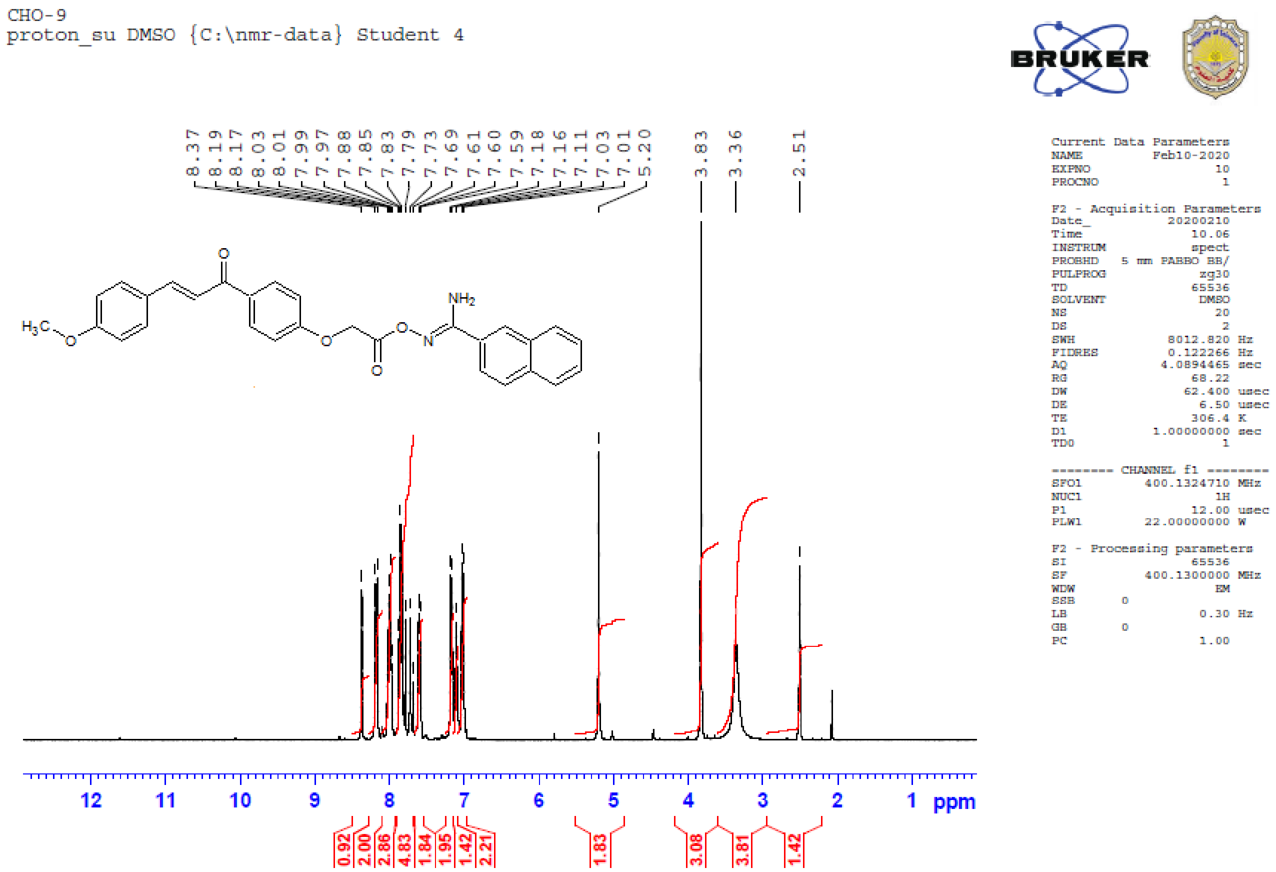

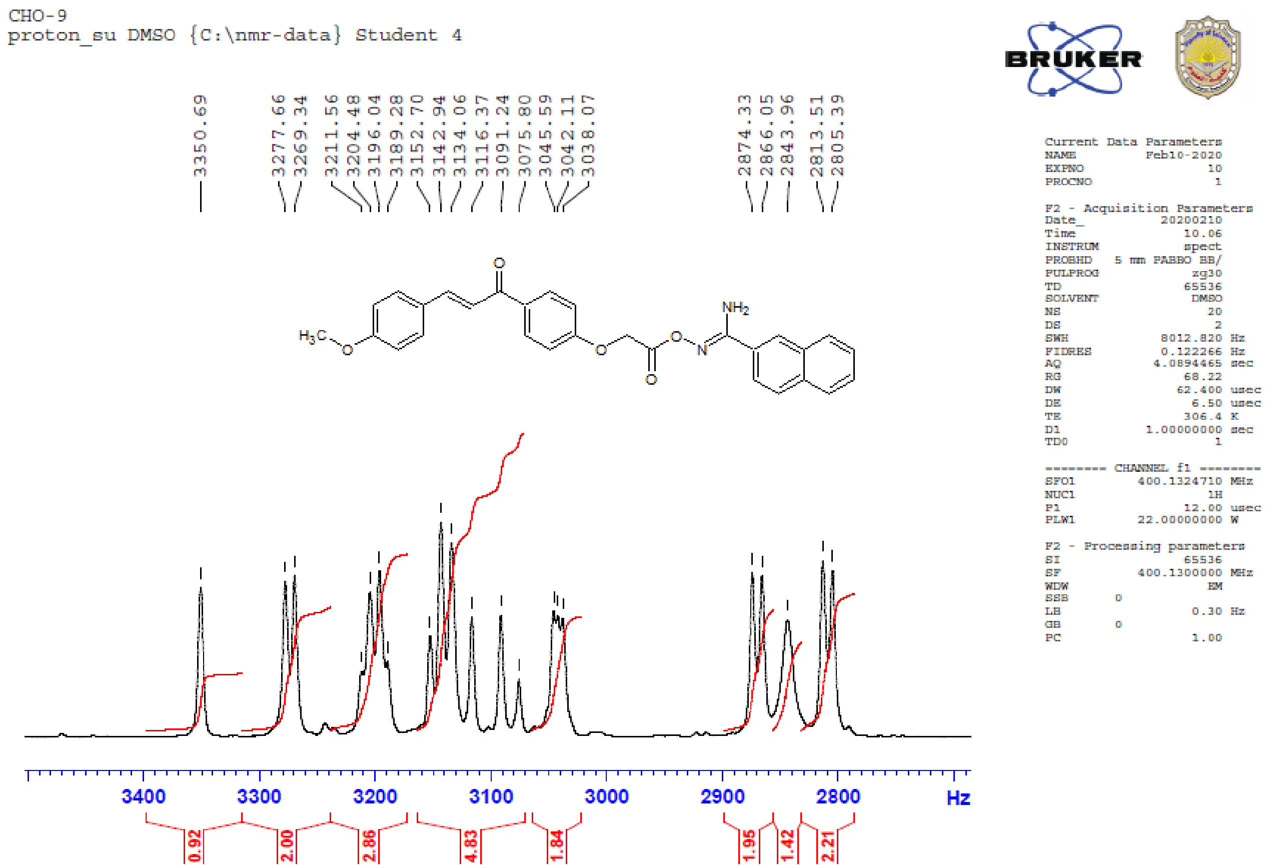

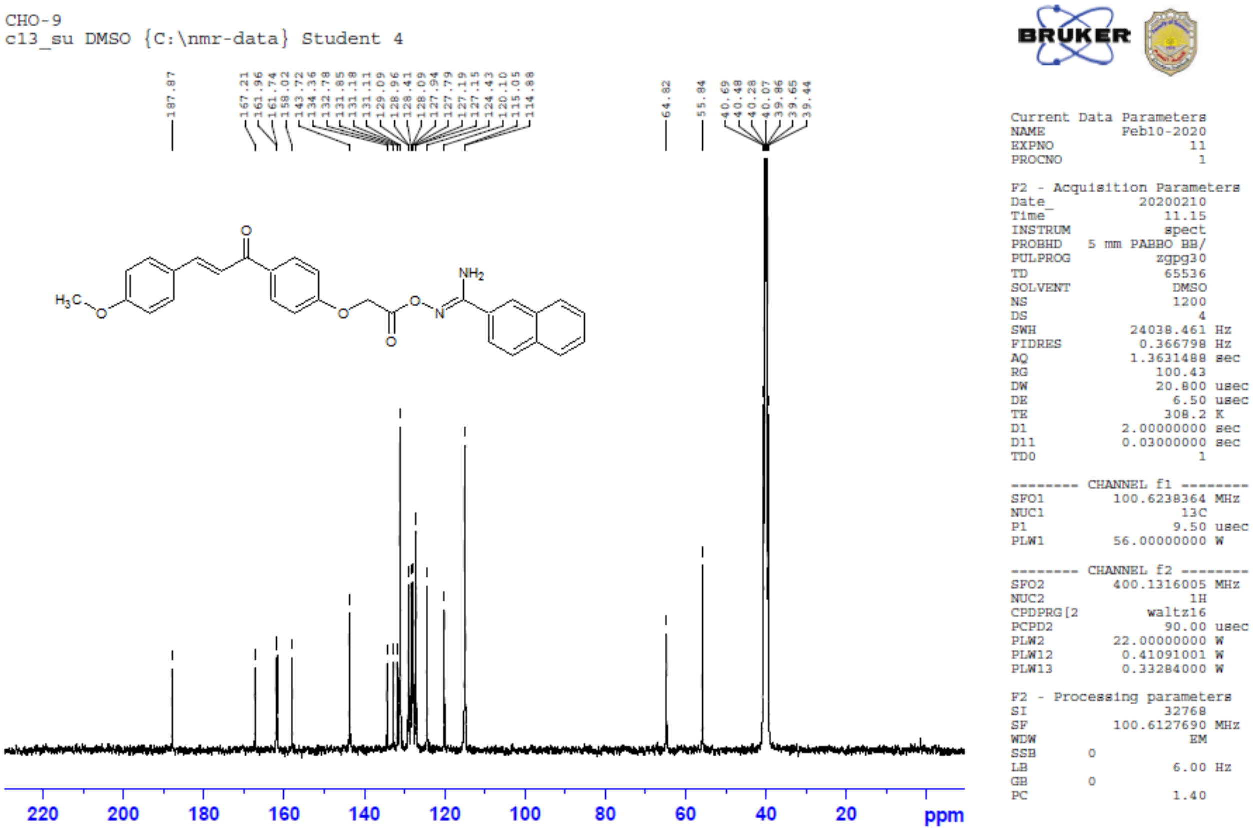

^{1}H and ^{13}C NMR Spectra of 6d:

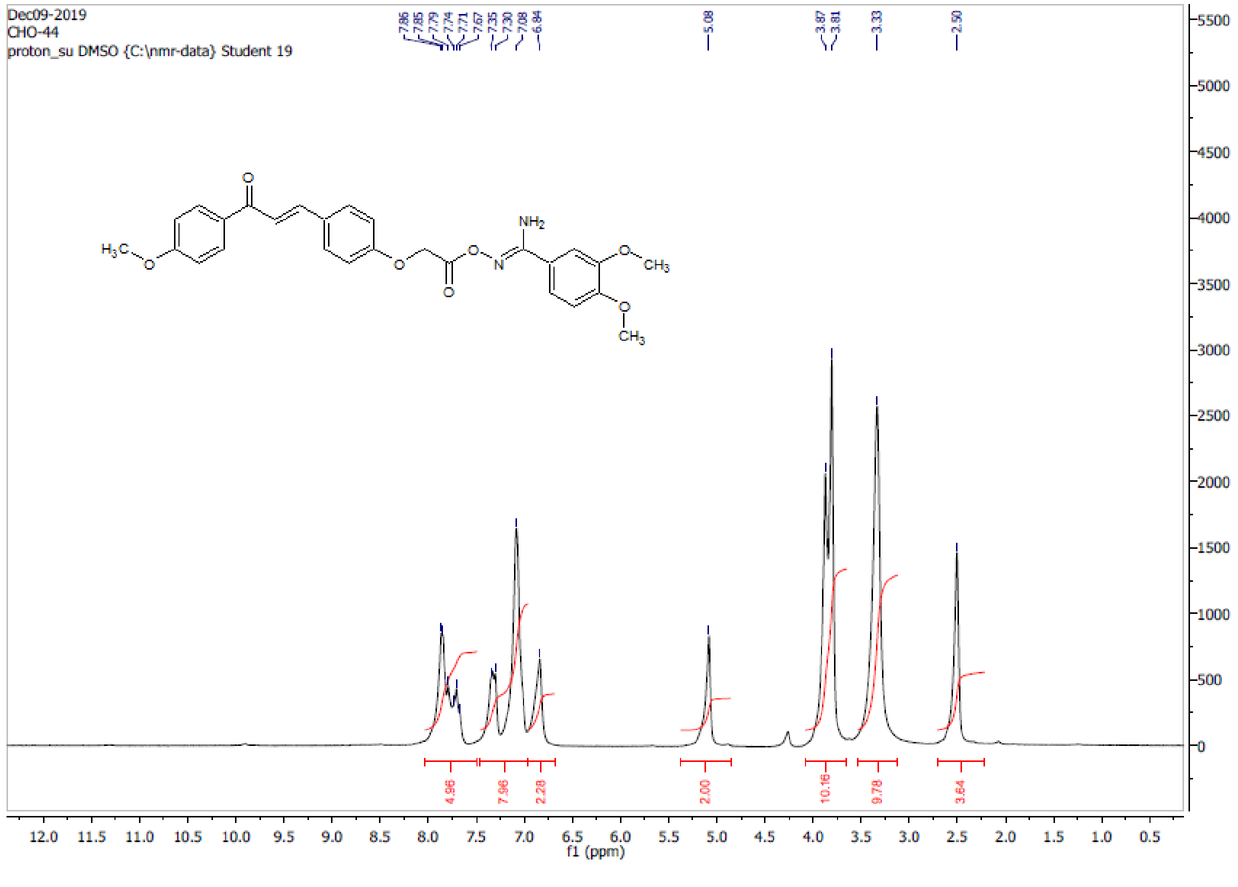

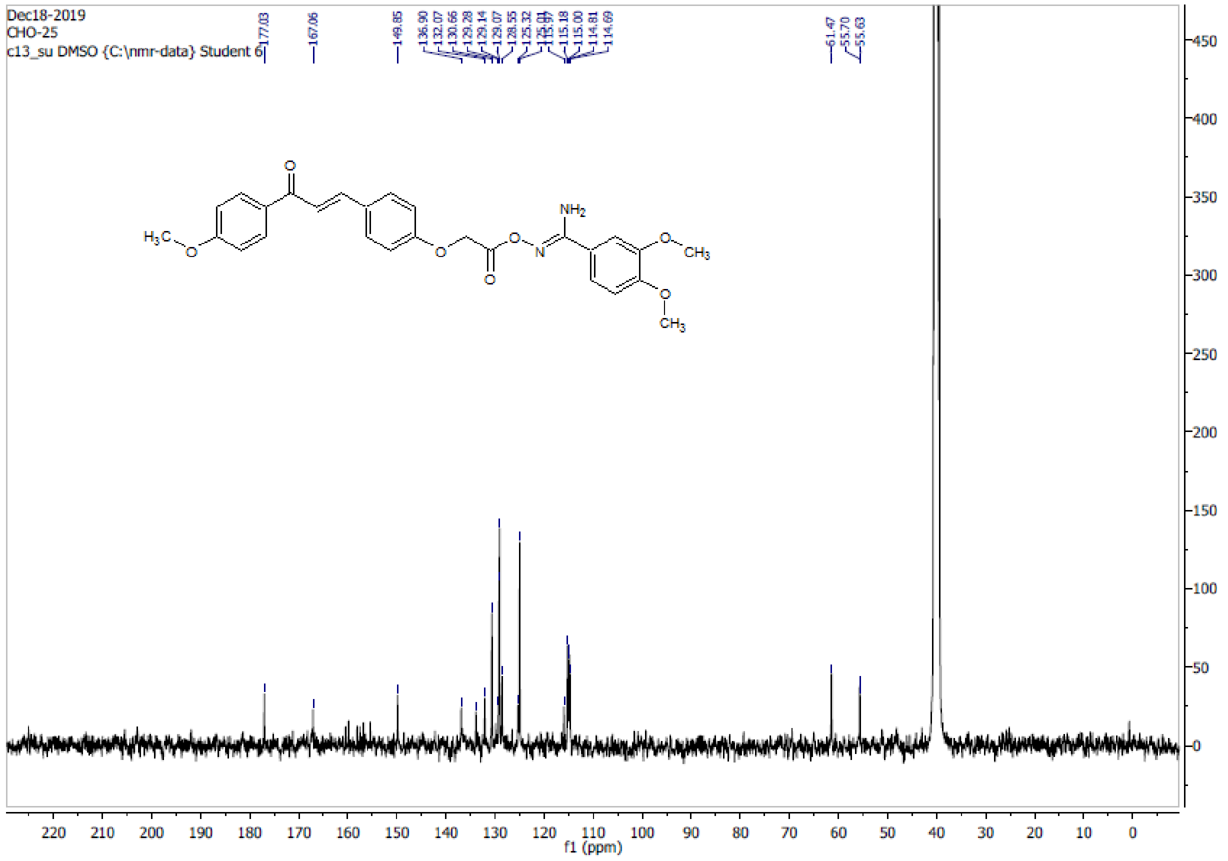

Mass Analysis Report

Mass Interface used: TLC

2) Experimental:

A) Device Specification: Advion compact mass spectrometer (CMS) NY | USA is provided with the following specification:

Ion Source: ESI, APCI, or APCI/ASAP

Polarity: Positive & negative ion switching in a single analysis

Flow Rate Range:

- ESI: 10 µL/min to 1 mL/min
- APCI: 10 µL/min to 2 mL/min

m/z Range:

- Expression S m/z 10 to 1,200
- Expression L m/z 10 to 2,000

Acquisition Speed: 10,000 m/z units/sec

Sensitivity (ESI): 10 pg reserpine (FIA – 5 µL injection at 100 µL/min) 100:1 S/N (RMS) with SIM of m/z 609.3

Accuracy: ±0.1 m/z units of the entire acquisition range

Stability: 0.1 m/z units at m/z 1,200 over 12 hour period operating temperature of 20ºC ±1ºC

Polarity Switching Speed: 50 ms

Dynamic Range: 4.5 orders of magnitude.

B) Method:

• Sample preparation: samples were dissolved in methanol and spotted on a TLC plate before introduction to the device

• Adjustment parameters: Mode of fragmentation: Typical

• Mass Range from 100 to 1200 • Mass type: ESI

• N.B standard querciten (mol wt= 302) was injected to assure the quality of analysis.

4a

- ESI-MS (*m/z*): 429.4, [M-H]$^-$

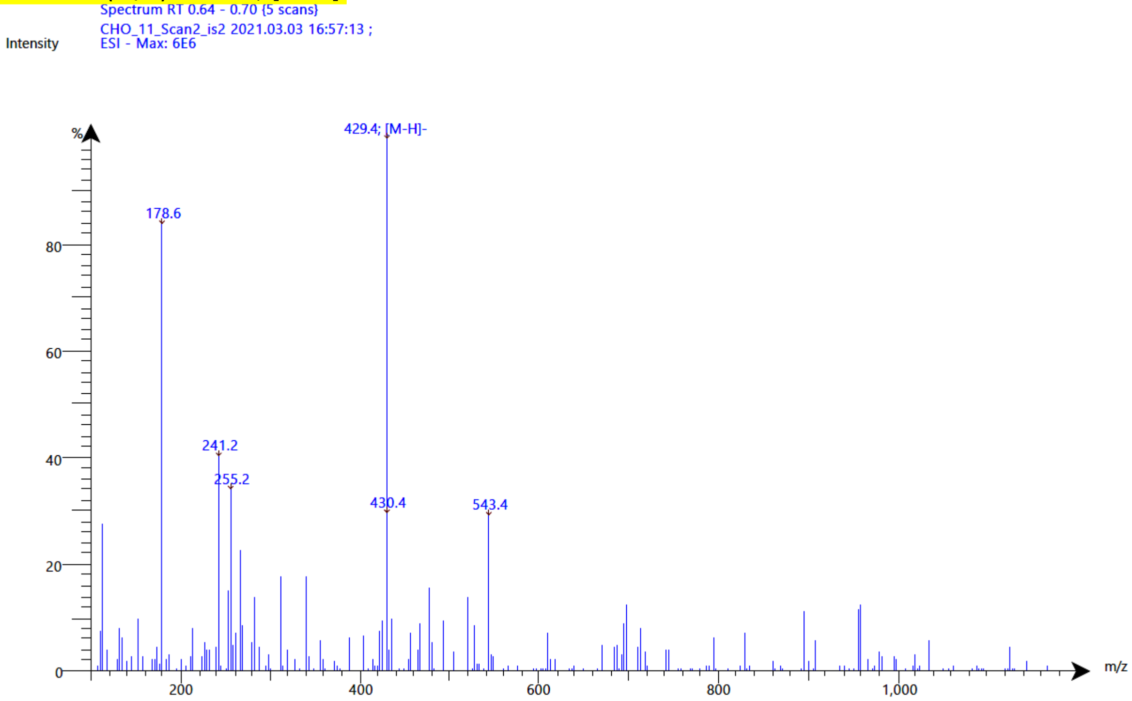

Fig. S1. Negative ESI-MS: precursor ion at *m/z* 429.4, [M-H]$^-$, corresponds 4a.

4b

- ESI-MS (*m/z*): 463.3, [M-H]$^-$

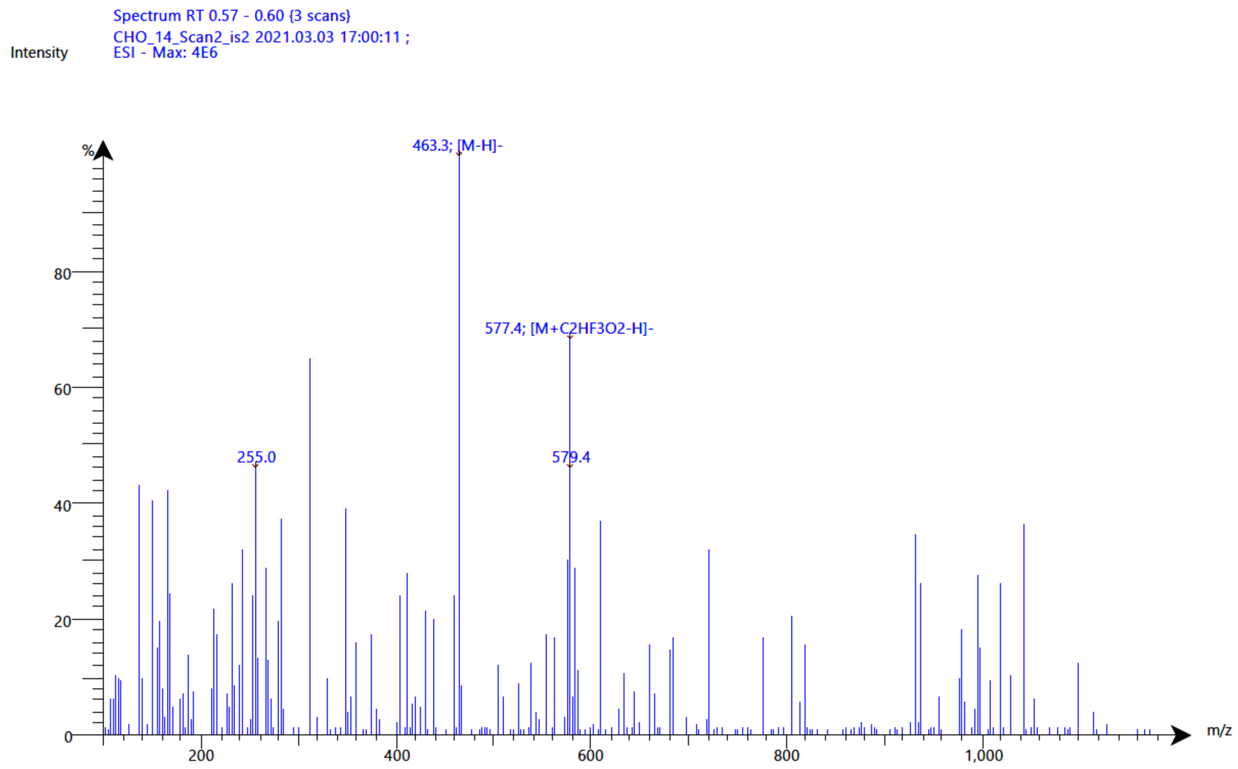

4f

-ESI-MS (*m/z*): 479.3, [M-H]$^-$

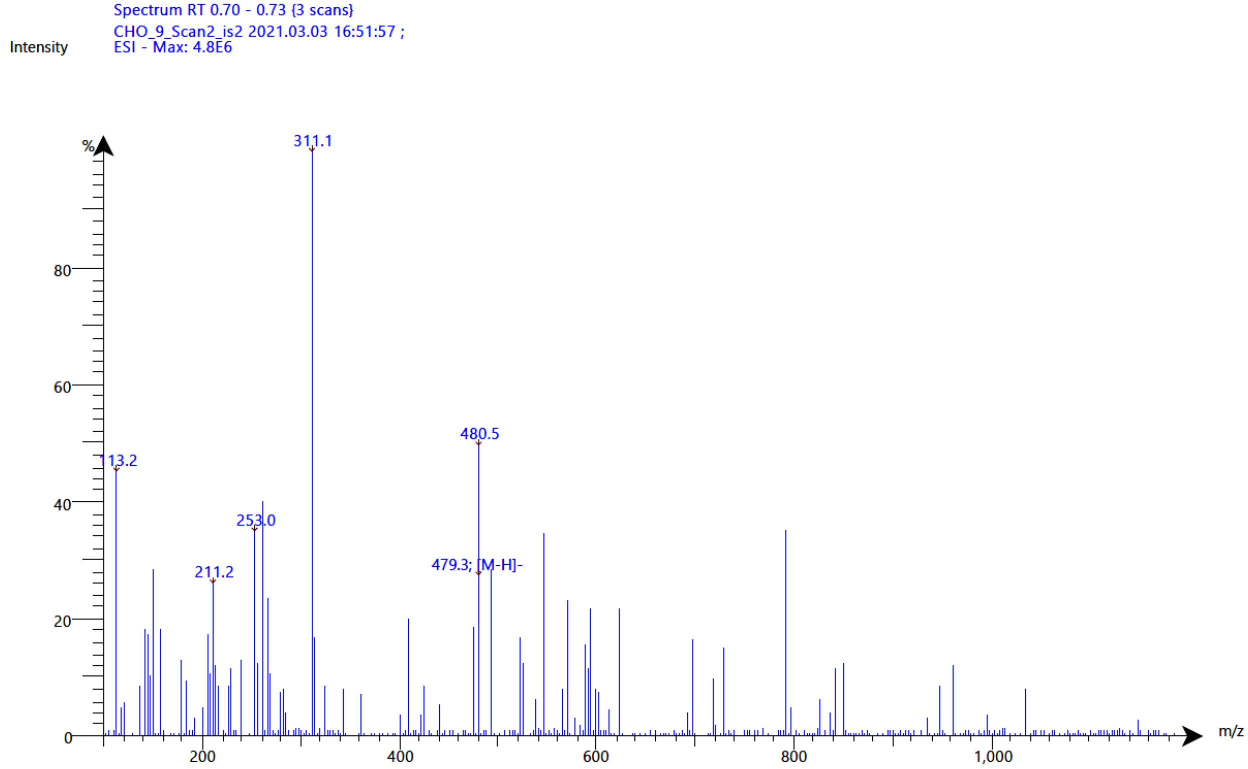

6a

-ESI-MS (*m/z*): 429.3, [M-H]$^-$

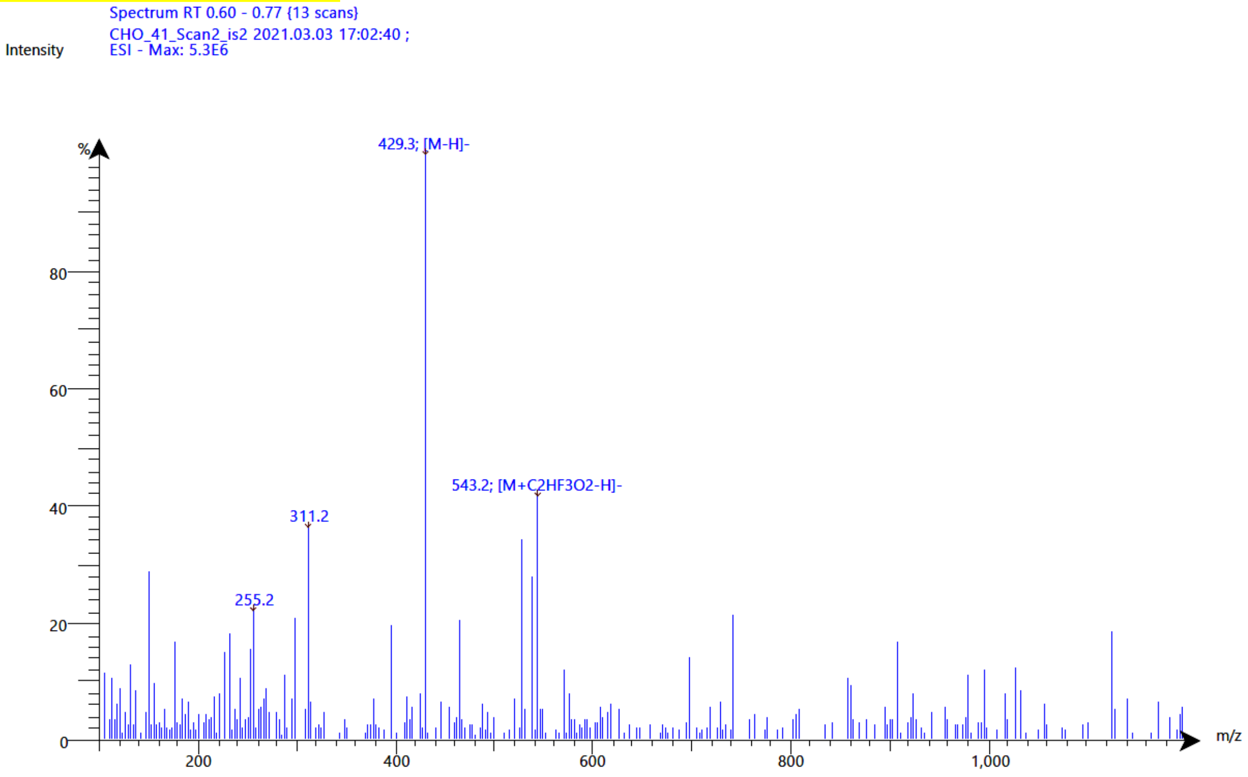

6d

-ESI-MS (*m/z*): 489.5, [M-H]$^-$

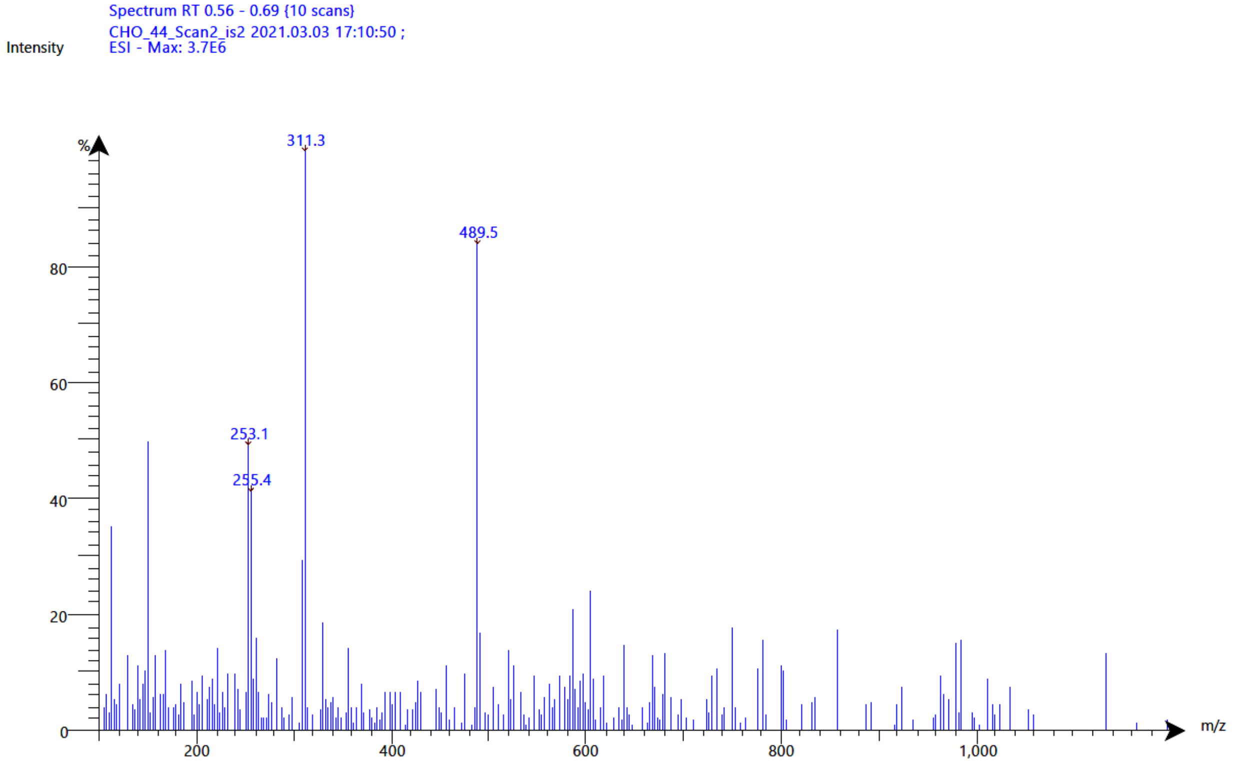

6e

-ESI-MS (*m/z*): 474.4, [M-H]$^-$

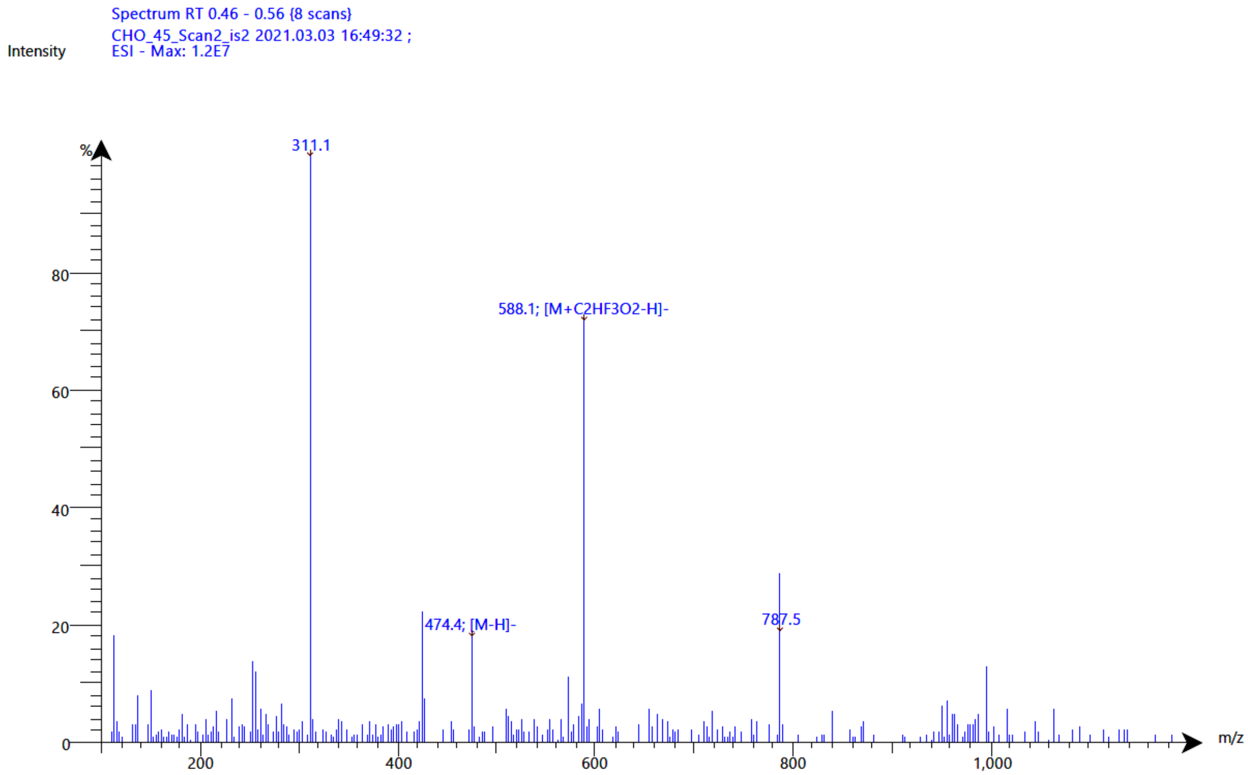

6f

-ESI-MS (*m/z*): 479.2, [M-H]⁻

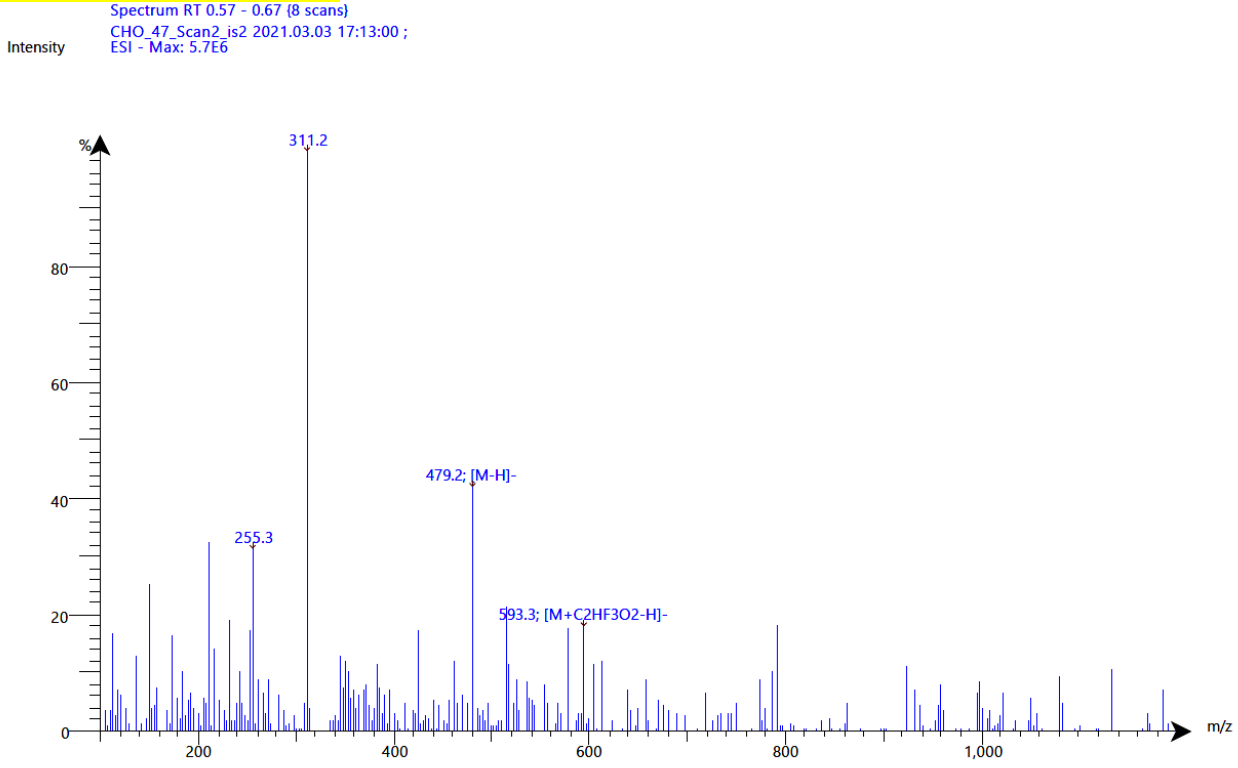

Supplement: Supplemental Material [file IENZ_A_1929201_SM4024.pdf]
